# Effectiveness of Surgical Face Masks in Reducing Acute Respiratory Infections in Non-Healthcare Settings: A Systematic Review and Meta-Analysis

**DOI:** 10.3389/fmed.2020.564280

**Published:** 2020-09-25

**Authors:** Min Xian Wang, Sylvia Xiao Wei Gwee, Pearleen Ee Yong Chua, Junxiong Pang

**Affiliations:** ^1^Saw Swee Hock School of Public Health, National University of Singapore and National University Health System, Singapore, Singapore; ^2^Centre for Infectious Disease Epidemiology and Research, National University of Singapore, Singapore, Singapore

**Keywords:** surgical mask, systematic review, acute respiratory infection, non-healthcare settings, prevention

## Abstract

**Background:** Acute respiratory illnesses (ARIs) are the most common respiratory infectious diseases among humans globally. Surgical mask (SM) wearing has been shown to be effective in reducing ARI among healthcare workers. However, the effectiveness of SM in reducing ARI in the non-healthcare settings remains unclear. This review aims to summarize and assess the association between SM wearing and ARI incidence, from existing interventional and observational studies conducted in non-healthcare settings.

**Methods:** Systematic literature searches conducted in PubMed, Cochrane Library, and Embase databases identified 503 unique studies. After screening, 15 studies (5 randomized controlled trials and 10 observational studies) were assessed for reporting and methodological qualities. Proportions of ARI episodes in each group and adjusted summary statistics with their relevant 95% CIs were extracted. Data from 10 observational studies were pooled using the generic inverse variance method.

**Results:** A total of 23,892 participants between 7 and 89 years old involved across 15 studies from 11 countries were involved. Key settings identified were Hajj, schools, and in-flight settings. A modest but non-significant protective effect of SM on ARI incidence was observed (pooled OR 0.96, 95% CI 0.8–1.15). Subgroup analysis according to age group, outcome ascertainment and different non-healthcare settings also revealed no significant associations between SM use and ARI incidence.

**Conclusion:** Surgical mask wearing among individuals in non-healthcare settings is not significantly associated with reduction in ARI incidence in this meta-review.

## Introduction

Acute respiratory infections (ARIs) have resulted in significant morbidity and mortality globally. Many respiratory viruses attribute to ARI. These include influenza viruses, rhinoviruses, and coronaviruses. Coronaviruses, namely, human coronavirus NL63, 229E, OC43, and HKU1, attributed to a significant proportion of ARI (1, 2). Similarly, SARS-CoV (2003) (3), MERS-CoV (2012) (4), and the recent SARS-CoV-2 (5) are transmitted via droplet/aerosols and close contacts and resulted in significant fatality. At the time of writing, the global toll of COVID-19 stands at 2,145,512 cases, including 143,308 deaths (6).

In the absence of pharmaceutical interventions such as vaccine and anti-virals for most respiratory viruses including coronavirus disease 2019 (COVID-19) (7), non-pharmaceutical interventions such as personal protection equipment are crucial to curb community spread (7). However, there are inconsistent policies and recommendations on the use of surgical masks (SM) in the community in the early stage of the COVID-19 pandemic. WHO (8), Centers for Disease Control and Prevention (CDC), and national authorities have advocated the usage of SM, as opposed to N95 respirators, only among symptomatic individuals. Otherwise, one is to practice good personal and hand hygiene as the key mitigation measure.

WHO only conditionally recommends SM wearing by asymptomatic individuals in the community in situations of epidemic and pandemic (9). However, as community transmission becomes more rampant in many countries at the early phase of the pandemic, mask wearing has become a norm, as asymptomatic transmission remains a possibility with limited evidence to show otherwise (10, 11). With an increase in SM usage worldwide, a global shortage which is detrimental to the healthcare setting and pandemic control ensues.

The efficacy of SM usage to prevent transmission of influenza-like illness (ILI) and laboratory-confirmed influenza have been shown in a number of studies among symptomatic patients (12–14). However, the protective effect of SMs among healthy individuals in a community setting remains unclear. Existing systematic reviews and meta-analyses consistently found SMs ineffective at preventing ILI or influenza episodes when worn by an uninfected individual (15–17). However, a study that examined the protective effect of SM use against secondary influenza episode in a household setting, found a 70% reduction in reported episodes when participants were compliant in SM use (18).

Conflicting stance regarding the usage of SMs among healthy individuals to reduce the risk of respiratory infections remains even with the publication of a systematic review assessing the efficacy/effectiveness of SM against respiratory infections in 2011 (16). The review found face mask to be the best performing non-pharmaceutical intervention across seven included studies. However, all included studies primarily assessed SARS incidence only, and were predominantly hospital based (85.7%) or only involved healthcare workers (71.4%). The remaining non-hospital-based study involved SM usage in households with healthcare workers as the index case, and another included non-healthcare workers who were hospitalized. In hospital settings and/or among healthcare workers, occupational requirements, and increased knowledge on personal protection increase compliance to SM usage. However, SM usage may differ significantly in non-healthcare-related settings or workers. Thus, the review's findings may not extend to a community setting and/or a non-healthcare setting. With the limited supply of surgical mask for the healthcare workers globally to manage the large influx of COVID-19 patients, there is a pressing need to investigate the efficacy or effectiveness of SM use in the non-healthcare settings so as to guide policyholder on the usage of SM in the community. Thus, the study aims to perform a systematic review and meta-analysis to assess the effectiveness or efficacy of SM usage in decreasing the incidence of respiratory infectious disease and non-influenza respiratory infection in the community.

## Materials and Methods

### Search Identification and Selection

Systematic literature searches were conducted in PubMed, Cochrane Library, and Embase databases. Eligible studies were assessed for the reporting and methodological quality. Proportions of populations reporting ARI episodes in each group and adjusted summary statistics with their relevant 95% CIs were extracted when reported. A pooled odds ratio was estimated using the generic inverse variance method and heterogeneity was assessed. Relevant peer-reviewed literature that assessed the effectiveness/efficacy of surgical face masks (SM) in preventing community-acquired acute respiratory infections (ARIs) were identified and extracted from PubMed, EMBASE, and Cochrane databases on February 25, 2020. Specific search terms defined by the Population, Intervention/Exposure, Comparator, and Study design (PICOS/PEOS; Supplementary Table 1) utilized for each database are provided in (Supplementary Table 2). In all databases, a filter to identify studies published from 2010 was applied to capture more recent published studies that are more representative of the current social, behavioral, educational, and economic status of the general population, which may be attribute to the risk of ARI and compliance to SM usage. Reference lists of relevant reviews were also hand-searched to identify additional studies. This study was conducted in accordance to Cochrane's Preferred Reporting Items for Systematic Reviews and Meta-Analyses (PRISMA) guidelines.

Identified publications were screened according to criteria in the following hierarchy by three authors, and any disagreement was reviewed by the fourth author to reach a final consensus, and included in the review if they fulfilled all criteria:

- Type of intervention/exposure: Surgical face mask usage in comparison with a comparable control group (no surgical face mask usage or use of hand hygiene practices only).
- Type of study: Peer-reviewed publications on interventional (randomized controlled trials) and observational (cohort studies, cross-sectional studies, and case-controlled studies) studies.
- Type of participants: Participants are individuals living in a general community setting, not healthcare workers or patients in clinical and medical setting.
- Types of outcomes: Incidence or episodes of (i) acute respiratory infectious disease and (ii) non-influenza respiratory infections in a community setting.

**Figure 1:**
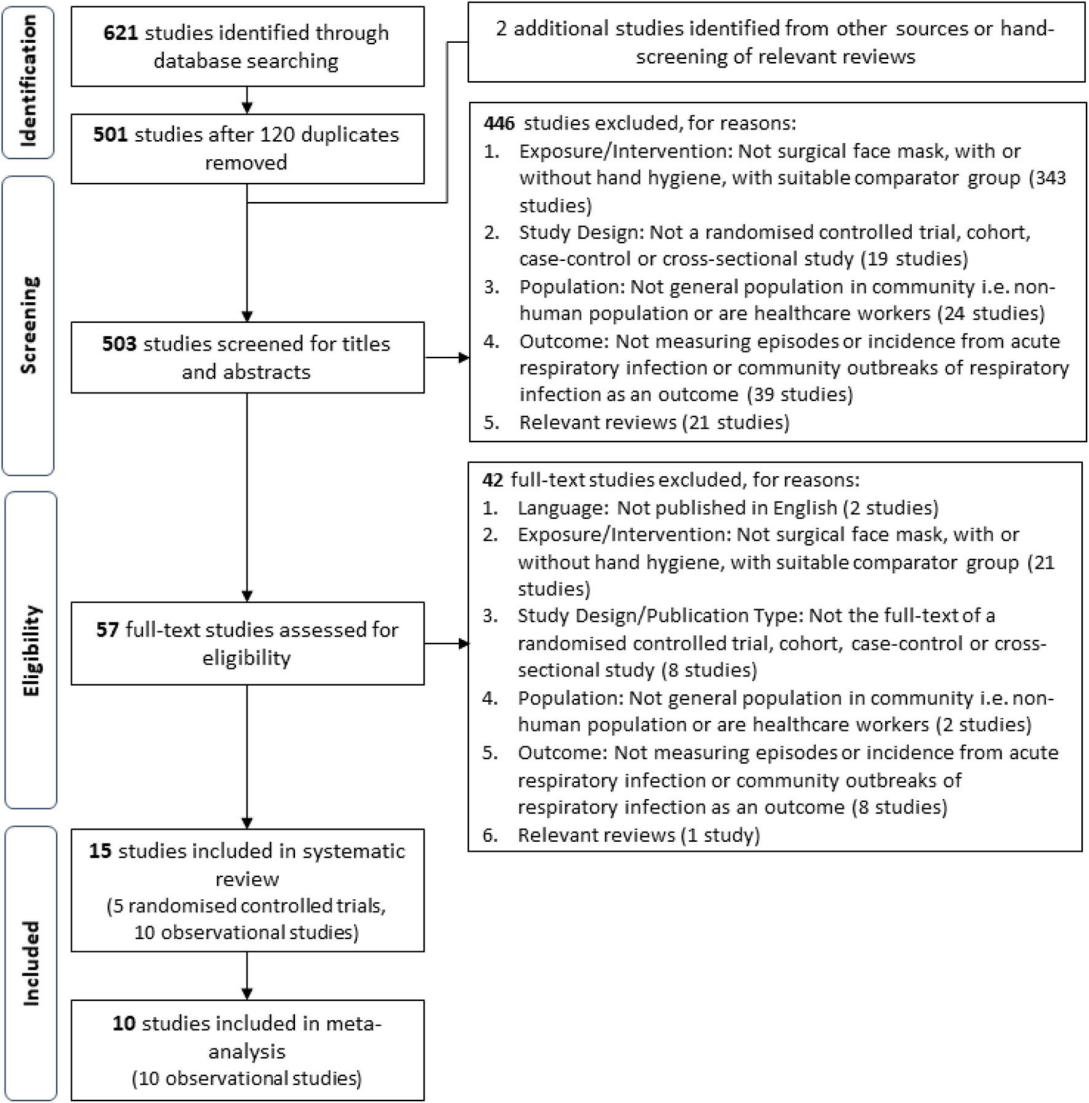
Flowchart of the process of screening and study selection.

This review defines ARI as any acute respiratory infectious disease, including influenza-like illnesses and non-influenza respiratory infections, regardless whether the illness was clinically diagnosed, laboratory confirmed, or self-reported as defined by the study. Studies utilizing variations of facial protective gear (e.g., respirators and goggles) as an intervention/exposure or were conducted in settings outside of the general community were excluded in alignment with the goal to assess the recommendation of SM usage in a community setting. Relevance of the extracted studies was first assessed with titles and abstracts, before full texts of relevant studies were retrieved for further screening and validation based on the aforementioned criteria. A PRISMA flow diagram of the study selection process is shown in Figure 1.

### Data Extraction

Data extracted from included studies were consolidated with Microsoft Excel 2016, and presented in Tables 1, 2. Corresponding authors of included studies were contacted when clarification or more information were required. The following data were extracted from each study: authors, year of publication, study and population characteristics, description of the measures implemented in intervention and control groups, and outcomes. Study designs of included studies were also assessed based on their design features as recommended by the Cochrane Handbook for Systematic Reviews and Meta-analyses (33), in addition to extracting their reported study design. Outcome measures extracted for the intervention and control groups, when available, include (1) number of ARI episodes, (2) summary statistic for ARI incidence [relative risk (RR), odds ratio (OR), or hazard ratio (HR)] and their corresponding 95% confidence interval (95% CI), and (3) any other key findings.

**Table 1:** Characteristics of included studies.

**References**	**Study design**	**Country of study; study duration**	**Population size (% men); percentage with recent flu vaccine**			**Age group, mean age (SD) in years; description of population health status**	**Description of measures implemented**		**Overall quality**	
			**Overall**	**Control**	**Intervention**		**Control**	**Intervention**	**Reporting**	**Methods**
**Randomized controlled trials**										
Aiello et al. (19)	Cluster randomized trial	USA; 6 weeks	930 (26.7%); 13.8%	552 (18.1%); 14.7%	378 (39.2%); 12.4%	Adult; 18.7 (0.8); Participants are seemingly healthy college students from University of Michigan during the 2006–2007 influenza season	Basic hand hygiene education	Wearing surgical facemask as much as possible in residence hall during intervention period	High	High
Simmerman et al. (20)	Cluster randomized trial	Thailand; 1 week	583 (40.5%); 0%	292 (40.1%); 0%	291 (40.9%); 0%	Adult; 34 (24–42)^*^; Household contacts of a pediatric index case with influenza-like illness, health status of participants not reported	Hand washing education and kit	Mask wearing in index patients and all household contacts from any point in time within 7 days from randomization, and hand washing education and kit	High	High
Aiello (2012) (21)	Cluster randomized trial	USA; 6 weeks	762 (43.0%); 16.3%	369 (43.9%); 17.6%	391; (42.5%); 15.1%	Adult; 18.95 (0.9); Participants are seemingly healthy college students from University of Michigan during the 2007–2008 influenza season	Basic education on proper hand hygiene and use of standard surgical face masks	Wearing surgical facemask for ≥6 h in residence hall during intervention period	High	High
Suess et al. (18)	Cluster randomized trial	Germany; 8 days	151 (48.3%); 9.3%	82 (47.6%); 7.3%	69 (49.3); 11.6%	Adult and child Control^*^: 2009: 35 (18–40) 2010: 38 (12–43) Intervention^*^: 2009:37 (12–43) 2010: 35 (17–42) Household members of laboratory-confirmed index patients during 2 consecutive influenza seasons (Nov 2009–Jan 2010 and Jan–Apr 2011) in Berlin. Chronic illness was present in household members in the following proportions: 19.8% of the control group and 15.4% of the intervention group	Provision of general information on infection control to household	Healthy household members to wear masks at all times when in one room with the index patient and/or any other household member with respiratory symptoms	High	High
Barasheed et al. (22)	Cluster randomized trial	Australia; 1 week	89 (NR); NR	53 (NR); NR	36 (NR); NR	Adult and child Control: 41.6 (17–72)^*^ Intervention: 48 (19–80)^*^ Australians attending Hajj in 2011, health status of the pilgrims were not reported but 36 of them were aged 65 and above or had chronic disease	No face masks provided; only general information on hygiene was provided	Provision of face masks, and advice and instructions on mask usage through participants' stay in Mina	Low	High
**Observational studies**										
Deris et al. (23)	Cross-sectional	Malaysia; entire hajj duration	387 (56.6%); 72.9%	105 (NR); NR	282 (NR); NR	Adult; 50.4 (11); Health status of Malaysian pilgrims attending Hajj in 2007 was not reported	Non-usage of face masks during Hajj	Use of face mask during Hajj	Low	
Gautret et al. (24)	Cohort	France; 4 weeks	274 (NR); NR	56 (NR); NR	218 (NR); NR	Adult; 58 (23–83)^*^; French pilgrims attending Hajj in 2009 had varying health status: 23.7% had diabetes mellitus, 5.5% had chronic respiratory disease, 3.3% had chronic cardiac disease, and 2.2% had other chronic conditions	Non-usage of face masks during Hajj	Use of face mask during Hajj	Low	
Al-Jasser et al. (25)	Cross-sectional	Saudi Arabia; 2 weeks	1,507 (61/7%); 94.4%	328 (NR); NR	216 (NR); NR	Adult; 37.9 (12.2); Hajj pilgrims living in Riyadh city who were performing Hajj in 2010, 18.4% of the pilgrims had chronic diseases including diabetes, hypertension, cardiac diseases, bronchial asthma and renal diseases	Never used face mask during Hajj in Mecca	Use of face mask most of the time during Hajj in Mecca	High	
Al-Jasser et al. (25)				328 (NR); NR	635 (NR); NR		Never used face mask during Hajj in Mecca	Use of face mask sometimes or occasionally during Hajj in Mecca		
Balaban et al. (26)	Cross-sectional	USA; 24.1 weeks^*^	186 (49.5%); at least 74.2%^#^	54 (NR); NR	89 (NR); NR	Adult and child; 48.9 (16–89)^#;^ US pilgrims attending the 2009 Hajj, who resided in Michigan and Minnesota, of which 31 pilgrims were with chronic conditions (diabetes, hypertension, asthma), of which 13 were <65 years old	Non-usage of face masks during Hajj	Use of face mask during Hajj	High	
Kim et al. (27)	Cross-sectional	South Korea; 3 weeks	7,449 (42.3%); 23.1%	2,082 (NR); NR	466 (NR); NR	Child; 12.97 (3.03); School-aged children between 7 and 18 years old, attending schools in Seodaemun-gu, Seoul, some children had the following conditions: asthma (*n* = 171), atopy (*n* = 891), cardiac disease (*n* = 20), renal disease (*n* = 12), liver disease (*n* = 11), diabetes (*n* = 6)	Non-usage of face mask	Continued use of face mask	High	
Kim et al. (27)				2,082 (NR); NR	2,819 (NR); NR		Non-usage of face mask	Irregular use of face mask		
Gautret et al. (28)	Cross-sectional	France; 3 consecutive Hajj seasons	360 (NR); 31.6%∧	167 (NR); NR	193 (NR); NR	Adult; 60.6 (22–85)^#;^ French pilgrims attending Hajj from 2012 to 2014 were with the following comorbidities: 55.1% had a chronic disease; 30.2% with hypertension, 27.5% with diabetes, 8.4% with chronic cardiac disease, 7.6% with chronic respiratory disease, 1.3% with immune deficiency, and 0.3% with chronic renal disease	Non-usage of face masks during Hajj	Use of face mask during Hajj	Low	
Hashim et al. (29)	Cross-sectional	Malaysia; 1 week	468 (56.2%); 65.2%	80 (NR); NR	322 (NR); NR	Adult and child; 52.52 (10.15); 60% of the Malaysian pilgrims attending the 2013 Hajj had at least one medical illness: 26.5% had hypertension, 15.4% had diabetes mellitus, 9.0% had allergic rhinitis, 5.6% had bronchial asthma and others (3.6%)	Non-usage of face masks during Hajj	Use of face mask during Hajj	High	
Uchida et al. (30)	Cross-sectional	Japan; 1 week	10,524 (51%); 48.1%	5,050 (NR); NR	5,474 (NR); NR	Children; 9.45 (7–12)^#^; 10.6% of the schoolchildren recruited from elementary schools in Matumoto City had underlying diseases	Non-usage of face masks	Use of face mask at any place or time during the 2014/2015 influenza season (response provided by guardians of the children)	High	
Emamian et al. (31)	Nested case–control	Saudi Arabia; During Hajj	95 (57.9%); 75.8%	38 (NR); NR	57 (NR); NR	Adult; NR, but 54.7% of the pilgrims were <60 years and 47.3% were ≥60 years old; 38.95% of recruited Hajj pilgrims had at least systemic disease, defined as asthma, diabetes mellitus, hypertension, chronic obstructive pulmonary disorder and cardiovascular diseases)	Non-usage of face masks during Hajj	Use of face mask during Hajj	Low	
Zhang et al. (32)	Retrospective case–control	China; 2 weeks	41 (48.8%); NR	26 (NR); NR	15 (NR); NR	Adult and child; Not reported but age demographics are as follows: 19.5% were <20 years old, 46.3% were between 20 and 40 years old, and 34.2% were >40 years old; Health status of passengers on the flight from New York to Hong Kong, including a stopover in Vancouver, was not reported	Non-usage of face masks during any leg of the flight	Use of face mask during either leg of the flight: (1) New York to Vancouver, (2) Vancouver to Hong Kong, (3) New York to Hong Kong, and (4) Hong Kong to Fuzhou	Low	

* *Median (interquartile range)*;

# *mean (range)*.

**Table 2:** Key findings of wearing surgical face masks on ARI incidence.

**References**	**Outcome measured and ascertainment**	**Outcome definition**	**% Population infected (infected/population size)**		**Reported summary risk estimate: risk ratio (RR), odds ratio (OR), or hazard ratio (HR) (95% CI);** ***p*****-value (if significant)**			**Key findings**
			**Control**	**Intervention**	**RR**	**OR**	**HR**	
Aiello et al. (19)	Self-reported ILI through weekly survey on ILI symptoms, and clinical diagnosis of ILI by study nurse during scheduled visits	ILI defined as presence of cough and at least 1 constitutional symptom (fever/feverishness, chills, or body aches)	32.1% (177/552)	26.2% (99/378)			0.9 (0.77–1.05)^a^	**Decreased but not statistically different ILI incidence rate** (*p* > 0.025) in face mask-only group, compared with control group from 4th week of intervention onwards Adjusted rate ratio^a^ (95% CI): 4th week: 0.72 (0.53–0.98) 5th week: 0.65 (0.42–0.98) 6th week: 0.58 (0.34–1.00)
Simmerman et al. (20)	Laboratory-confirmed secondary influenza episode	Positive rRT-PCR result on days 3 or 7 or a fourfold rise in influenza HI antibody titers with the virus type and subtype matching the index case	19.2% (58/302)	22.7% (66/291)				No significant difference in odds for secondary influenza infection in mask-wearing group compared with original control group provided with unrelated health education and no relevant non-pharmaceutical intervention Adjusted OR^b^ (95% CI) 1.16 (0.74–1.82) No significant difference in individual-level secondary attack rate across all experimental arms, including original control arm (Pearson χ^2^ for difference among the three intervention arms, adjusted for within-household correlation of 0.18 = 0.63)
Aiello et al. (21)	Self-reported ILI through weekly survey on ILI symptoms, and clinical diagnosis of ILI by study nurse during scheduled visits	Presence of cough and at least 1 constitutional symptom (fever/feverishness, chills, or body aches)	13.8% (51/302)	11.8% (46/291)			1.1 (0.88–1.38)^*c*^	No significant reductions in ILI or laboratory-confirmed influenza incidence in the face mask only group compared with the control, through the entire intervention duration, regardless whether summary estimates were adjusted^*c*^ or unadjusted
	Laboratory-confirmed influenza episode (only tested when ILI was self-reported/clinically diagnosed)	Positive RT-PCR result	4.3% (16/302)	3.1% (12/291)			0.92 (0.59–1.42)^c^	
Suess et al. (18)	Self-reported ILI, defined by the presence of fever with cough or sore throat	Presence of fever and cough or sore throat	34.1% (14/41)	17.1% (6/35)	0.61 (0.2–1.87)^d^			No significant difference in secondary attack rate across the groups, regardless whether the case was defined with ILI or laboratory-confirmed influenza definition, and after stratification for influenza season, virus subtype or timing of the first household visit (*p*-values ranged from 0.16 to 0.57) **70% reduction in odds of laboratory-confirmed influenza incidence** in mask-only group when per-protocol analysis was utilized Control OR (95% CI): reference Mask group OR^d^ (95% CI) 0.30 (0.1–0.94), p=0.04
								**Significant reduction in odds of laboratory-confirmed influenza incidence** in households who implemented intervention <36 h after symptom onset in index case. Intervention includes mask-only and mask and hand hygiene interventions in households Control OR (95% CI): reference Mask group + mask and hand hygiene group OR^e^ (95% CI) 0.16 (0.03–0.92), p=0.04
	Laboratory-confirmed influenza episode	Positive qRT-PCR result, with fever (>38.0), cough, or sore throat	46.3% (19/41)	17.6% (6/34)	0.39 (0.013–1.17)^d^			
Barasheed et al. (22)	ILI determined subjectively (questionnaire responses and symptom diaries) and objectively (results of testing on nasal swabs with point-of-care diagnostic test and nucleic acid tests)	Subjective or proven fever plus one respiratory symptom (dry/productive cough, runny nose, sore throat, shortness of breath), positive results in both point of care test (QuickVue A+B Influenza and Nucleic acid test for influenza and other respiratory viruses)	52.8% (28/53)	30.6% (11/36)^*^				Significantly lower ILI incidence in mask group than in control group (*p* = 0.04) Significantly lower rate of ILI in subjects who wear facemasks for >8 h compared with those who wear them for ≤ 8 h (*p* = 0.01) *Proportion with ILI symptoms in following group (%):* >8 h: 1/35 (2.9%) ≤ 8 h: 8/35 (22.9%)
Deris et al. (23)	Self-reported ILI during stay in Mecca, through a self-administered questionnaire	Triad of cough, sore throat, and fever (WHO definition)	32.4% (34/105)	42.9% (121/282)		1.57 (0.98–2.52)		No significant difference in ILI incidence in mask group than in control group (*p* > 0.05) **Significant difference** in sore throat incidence (OR 1.89; 95% CI 1.20–2.97, *p* = 0.006), and duration of sore throat and fever between mask wearing and control groups *Mean duration (SD) for mask vs. no mask:* Sore throat: 2.0 days (7.0) vs. 0.0 days (5.0), *p* = 0.008 Fever: 2.0 days (4.0) vs. 1.0 days (3.0), *p* = 0.039
Gautret et al. (24)	Self-reported ILI during their stay in Saudi Arabia andparticipation in the Hajj ritual, through a post-travel questionnaire	triad of cough, sore throat and fever (WHO definition)	3.6% (2/56)	9.2% (20/218)	2.57 (0.62–10.66)			No significant effect of preventive measures implemented on occurrence of cough, sore throat, rhinorrhea, voice failure, shortness of breath, and gastrointestinal symptoms during Hajj Preventive measures include vaccination, wearing a face mask, washing of hands, and use of hand disinfectants or disposable handkerchief
Al-Jasser et al. (25)	Self-reported URTI during Hajj in Makkah or within 2 weeks from return to Riyadh, through phone interview	Presence of at least one of the constitutional symptoms (fever, headache, myalgia) and one of the local symptoms (running nose, sneezing, throat pain, cough with or without sputum)	54.9% (180/328)	45.4% (98/216)	RR: 1.21 (1.03–1.42)^##^,^*^			**Significantly decreased risk** for URTI for those using face mask most of the time during Hajj, compared with those who never used it (*p* = 0.014) or only used it sometimes (0.045) during the Hajj **Significantly lower** URTI incidence in pilgrims who stayed at least 8 days in the Hajj area RR (95% CI) 0.78 (0.65–0.92), *p* = 0.006
Al-Jasser et al. (25)			55.2% (181/328)	53.7% (341/635)	RR: 1.17 (1.00–1.38) ^##^,^*^			
Balaban et al. (26)	Self-reported respiratory illness^f^ during their Hajj stay, through a telephone or in-person interviews within 14 days of pilgrims' return	Presence of one or more of the following localizing signs or symptoms: cough, congestion, sore throat, sneezing, or breathing problems	33.3% (18/54)	41.6% (37/89)		1.42 (0.70–2.88)^f^		No significant difference in odds for ILI incidence between the mask and control groups (*p* > 0.05) **Reduced odds** for ILI incidence when the social distancing, hand hygiene and contact avoidance were practiced *OR*^f^ *(95% CI) for the following protective behaviors:* Social distancing: 0.44 (0.22–0.90), *p* = 0.02 Hand hygiene: 0.36 (0.14–0.94), *p* = 0.03 Contact avoidance: 0.51 (0.24–1.11), *p* = 0.06
Kim et al. (27)	Laboratory-confirmed influenza A(H1N1) infection	Positive RT-PCR, influenza rapid antigen test, or viral cultures results	5.8% (120/2082)	3.0% (14/466)		0.51 (0.3–0.88)^*^		**Significant difference** in protective effects of facemask use and H1N1 infection (*p* = 0.004) **49% reduction** in odds for H1N1 infection with continuous facemask use, compared with occasional use or non-usage of facemasks
Kim et al. (27)			5.7% (119/2082)	5.8% (164/2819)		1.02 (0.83–1.25)^*^		
Gautret et al. (28)	Self-reported episodes of cough during Hajj travel, through a post-travel questionnaire	Presence of cough	78.4% (131/167)	81.9% (158/193)	RR: 1.04 (0.94–1.16)			No significant difference in cough prevalence between the mask wearing and control groups (*p* = 0.477) No significant effect of preventive measures practiced in reducing cough prevalence Preventive measures include frequent hand washing, use of hand sanitizer, disposable tissues or face mask, and influenza and/or invasive pneumococcal disease vaccination
Hashim et al. (29)	Self-reported respiratory illness^g^ while on Hajj in Saudi Arabia through pro-forma distributed before travel	Presence of at least one of the following respiratory symptoms: cough, subjective fever, or sore throat (non-ILI) or a triad of the listed symptoms (ILI)	NR	NR		1.65 (0.79–3.47)		No significant difference in odds for respiratory illness between the mask and control groups (*p* > 0.05) **Significant reduction** in odds for respiratory illness for groups with the following factors: previous experience of hajj or umrah, and those with good hand hygiene *OR*^g^ *(95% CI) for the following factors:* Previous Hajj experience: 0.24 (0.10–0.56) Previous umrah experience: 0.19 (0.07–0.52) Good hand hygiene: 0.35 (0.16–0.79)
Uchida et al. (30)	Clinically diagnosed influenza episode by physician, reported by child's guardian through questionnaire at the end of 2014/2015 influenza season	Seasonal influenza	21.4% (1080/5050)	19.5% (1069/5474)		0.86 (0.78–0.95)^*^^, 8^		Influenza incidence was associated with the following protective measures: mask wearing (*p* = 0.003), hand washing (*p* < 0.001), gargling (*p* < 0.001), and vaccination in this season (0.004) **Significant protective effect** of wearing a mask or vaccination during the influenza season against seasonal influenza incidence OR^h^ of vaccination during influenza season (95% CI) 0.87 (0.79–0.95), *p* = 0.004 **Increased odds** of seasonal influenza incidence when gargling or hand washing was practiced OR^h^, gargling (95% CI) 1.32 (1.18–1.47) OR^h^, hand washing (95% CI) 1.45 (1.27–1.64)
Emamian et al. (31)	Clinical diagnosis of respiratory tract infections by study staff, at point of entry to Mecca and Medina	All types of respiratory tract infections including tonsillitis, pharyngitis, laryngitis, sinusitis, otitis media, bronchitis, pneumonia, and influenza, except common cold	28.9% (11/38)	36.8% (21/57)		0.64 (0.23–1.78)		No significant difference in respiratory tract infections between mask wearing and control group (*p* > 0.05) No significant effect of other demographic variables or protective measures on odds of respiratory tract infection incidence Demographic variables include gender, age, years of education, room contact with other patients, room size, mean duration in holy places daily, body mass index, presence of systemic diseases, and smoking status Protective measures include mask usage, influenza vaccination status, salt water gargling, and use of personal prayer carpet
Zhang et al. (32)	Laboratory-confirmed influenza A(H1N1)pdm09 infection, through a post-travel telephone interview	Positive PCR result (RT-PCR and standard PCR) between 21 May to 4 June 2009	34.6% (9/26)	0% (0/15)		0.00 (0–0.71)^*^		**Decreased odds** of H1N1 infection in the mask group compared with the control group Significantly higher proportion of face mask usage in controls than in cases at all legs of the flight New York–Vancouver leg: *p* = 0.037 Vancouver–Hong Kong leg: *p* = 0.018 New York–Hong Kong leg: *p* = 0.018 Factors not associated to a case-passenger include exposure to any lavatories or specific lavatories, talking with other passengers, moving around the aircraft, and reported hand hygiene during the New York to Hong Kong leg (*p* > 0.05)

* *p < 0.05*.

## *Reference group was wearing mask most of the time*.

a *Adjusted for age, sex, race/ethnicity, handwashing practices at baseline, sleep quality, stress, alcohol consumption, and flu vaccination*.

b *Adjusted for household-level and individual-level characteristics (unspecified) in multivariable logistic regression analyses*.

c *Adjusted for gender, race, ethnicity, smoking status, physical activity, and having ever received a vaccination for influenza, intracluster correlation coefficient: −0.0005*.

d *Adjusted for age, sex, timely therapy of the index, vaccination of household contacts, time spent at home*.

e *Adjusted for age, sex, timely therapy of the index, time spent at home*.

f *Adjusted for (1) demographic and health factors (age, gender, education, whether respondent was US-born, health risk factors, seasonal influenza vaccination in the previous 12 months, influenza A(H1N1) vaccination before Hajj, and taking medication for respiratory illness during or post-Hajj), (2) travel-related factors (length of trip, international travel in the previous 12 months, and whether respondent had made a previous Hajj), and (3) influenza A(H1N1) knowledge and attitudes (if respondent received pre-travel health information, level of influenza A(H1N1) knowledge, perceived severity of influenza A(H1N1), and noticing influenza A(H1N1)-related health messages during the Hajj)*.

g *Adjusted for previous experience of Hajj or umrah, contact with pilgrims having respiratory illness and good practice of hand hygiene*.

h *Adjusted for gender, grade, underlying disease, sibling, regularly go out, vaccination in this season, mask wearing, hand washing, influenza in previous season*.

In addition, the review authors noticed some of the selected studies had an additional intervention group which implemented SM usage with hand hygiene practices in the general community. These studies were excluded from the main analysis, but the relevant data are also extracted and presented in Supplementary Table 8.

### Quality Assessment

Included studies were individually evaluated for their reporting and methodological quality using methods described in (Appendix B). For observational studies, reporting quality was evaluated using the STrengthening the Reporting of Observational Studies in Epidemiology (STROBE) statement (34, 35), and methodological quality was evaluated using the National Heart, Lung, and Blood Institute (NHLBI) quality assessment tool for Quality Assessment Tool for Observational Cohort, Cross-sectional and Case–Control Studies (36). For interventional studies, reporting, and methodology qualities were, respectively, assessed using the Consolidated Standards of Reporting Trials (CONSORT) statement (37), and Cochrane's Risk of Bias Tool (RoB 2.0) for cluster-randomized trials (38).

### Statistical Analysis

Pooled ORs with their corresponding 95% CIs were estimated with a random-effects model and the generic inverse variance method. The inbuilt RevMan calculator was used to estimate each study's OR and the corresponding 95% CIs when raw event data were available, otherwise the reported ORs were utilized. The estimated OR was subsequently utilized to calculate the log(OR) and standard errors of each individual study with the RevMan calculator. The I^2^ statistic and Cochran *Q*-test was used to evaluate statistical heterogeneity, where heterogeneity was characterized as minimal (<25%), low (25–50%), moderate (50–75%), or high (>75%) and was significant if *p* < 0.05. Subgroup analyses analyzing the effects of (1) study design (interventional vs. observational), (2) outcome ascertainment (self-reported or clinically diagnosed ARI episode vs. laboratory-confirmed ARI episode), (3) age, and (4) study setting (hajj setting vs. school setting vs. flight setting) on the protective effect of wearing SM on ARI incidence was also explored. Publication bias for studies included in the meta-analysis was assessed with conventional and contoured funnel plots. All statistical tests were two sided and performed using Review Manager 5.3, except for funnel plots that were generated with STATA 13 (StataCorp, Texas).

All stages of screening, data extraction, and study quality assessments were conducted in duplicate by MW, SG, and PC. Discrepancies were resolved by consensus with JP at the end of each procedure before moving on to the next stage of analysis.

## Results

### Screening Results and Characteristics of Included Studies

A total of 503 unique studies identified through our literature search were screened after the removal of 120 duplicates, and inclusion of two additional studies identified from external sources. The full texts of 57 potential studies were further assessed for eligibility and a total of 15 studies were selected for final inclusion into the review. The studies included in this systematic review are five cluster-randomized controlled trials (cluster RCT), seven cross-sectional studies, one cohort study, one nested case–control study, and one retrospective cohort study. All five RCTs and a cross-sectional study (29) were excluded from the meta-analysis. The reported summary statistics of three cluster RCTs were appropriately adjusted to account for the cluster design but were not the same measure [two studies reported HRs (19, 21), one study reported OR (18)]. Conventionally, only the same summary statistics across studies can be pooled using the generic inverse variance method when raw event data were unavailable. Hence, these reported summary statistics from these three cluster RCTs could not be pooled via the inverse generic variance method, whereas the remaining two cluster RCTs did not report summary statistics (20, 22). Thus, a total of 10 observational studies were included in the meta-analysis. The flow chart of the screening process and specific reasons for article exclusion are shown in Figure 1.

To provide readers with a general idea on the causal effect of SM usage on ARI incidence, the authors also explored combining the different summary measures reported using the generic inverse variance method (Figure 4). Summary statistics for all cluster RCTs were pooled after calculating the RRs for studies not reporting any summary statistics, with the RevMan calculator. Nonetheless, the authors did not consider the pooled estimate from cluster RCTs as part of the main meta-analysis results due to possible inaccuracy of the pooled estimate. Inaccuracy is likely present due to different summary measures across studies and the crude summary statistics [which were not adjusted for the clustering and other confounders present in the original study (39)] utilized to generate the pooled summary estimate.

An overview of the study characteristics is presented in Table 2. A total of 23,892 participants between 7 and 89 years old involved across 15 studies from 11 countries were included in this review. The health status of participants in all studies was all mixed, except for two studies which did not specify the health status of their participants (19, 21). Design features of seven studies suggested a retrospective cohort study design, although these studies were reported as cross-sectional (23, 25, 26, 29) or observational design (24, 30), or had no reported study design (28). The remaining eight studies had design features that corresponded to their reported study designs.

It is worthy to note that 8 of the 15 studies examined the effectiveness/efficacy of SM in hajj settings (22–26, 28, 29, 31) whereas the remaining investigated the same effect in students living on- or off-campus [4 studies (19, 21, 27, 30)], in households [2 studies (18, 20)], and in a flight setting [1 study (32)]. Of the five interventional studies included in this review, only one study compared hand washing with SM usage; the remaining four studies compared basic education—hand hygiene and/or SM usage and/or infection control–with mask wearing. The 10 observational studies included mainly compared the general lack of SM usage with its general use among participants; only 2 observational studies explored the effects with varying extents of SM usage on ARI prevention (25, 27).

### Systematic Review of Surgical Face Mask Wearing on ARI Incidence

Key findings on the effectiveness/efficacy of SM usage on ARI incidence are summarized in Table 3. Most studies assessed ARI incidence through self-reported influenza-like illness (ILI) as the sole (*n* = 4) or one of the outcomes together with laboratory-confirmed influenza (*n* = 2). The remaining studies assessed ARI incidence through laboratory-confirmed influenza (*n* = 3), clinically defined influenza (*n* = 1) or study-defined respiratory outcomes encompassing respiratory illness (*n* = 2), (upper) respiratory tract infections (*n* = 2), and cough (*n* = 1). A variety of summary risk estimates were reported when used, with seven studies reporting ORs, four studies reporting RRs, and two studies reporting HRs.

**Table 3:**
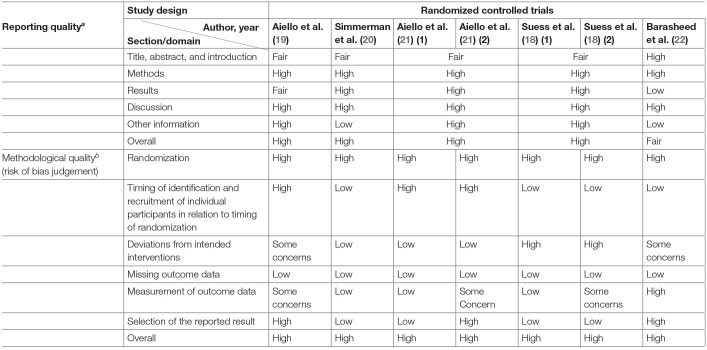
Summary of reporting and methodological quality of randomized controlled trials.

a *Reporting quality assessed with the Consolidated Standards of Reporting Trials (CONSORT) statement*.

b *Methodological quality assessed with the Cochrane's Risk of Bias Tool (RoB 2.0) for cluster-randomized trials*.

*Aiello et al. (21) (1) and Suess et al. (18) (1) assess risk of bias in the following outcome: laboratory-confirmed influenza episode. Aiello et al. (21) (2) assesses risk of bias in the following outcome: self-reported ILI episode. Suess et al. (18) (2) assesses risk of bias in the following outcome: clinically confirmed ILI episode*.

Across the studies, mixed effects of SM use on ARI incidence were observed, ranging from significantly decreased incidence (22, 25, 27, 30, 32) to no significant difference (18–21, 23, 24, 26, 28, 29, 31) compared with non-usage of SMs. Infection rates were generally lower in groups with SM usage, except in six studies (20, 23, 24, 26, 28, 31). Moreover, lower proportions of participants with ARI were consistently observed in groups who wore SMs for a longer duration [>8 vs. ≤ 8 h (22)] or more persistently [frequent/continued SM usage vs. occasional/irregular SM usage vs. non-SM usage (25, 27)] when studies stratified findings according to varying levels of SM usage. This suggests that varying extents of SM usage is associated with SMs' effectiveness in ARI prevention. Nonetheless, this difference in infection rates between groups were not significantly different (*p* > 0.05) in all but one study [*p* = 0.04 (22)].

It is worthy to note that when Suess et al. (18) analyzed data from compliant participants (i.e., per-protocol analysis), only a 70% reduction in odds of laboratory-confirmed secondary influenza episode was observed among household members with SM usage compared with household members without it (OR 0.30, 95% CI 0.10–0.94; *p* = 0.04) (18). A significant reduction in odds of laboratory-confirmed secondary influenza incidence was also observed in households who implemented interventions (used SM solely or in conjunction with hand hygiene practices) <36 h after symptom onset of the index case, regardless of participant compliance to the interventions (OR 0.16, 95% CI 0.03–0.92; *p* = 0.04).

### Meta-Analysis of Surgical Mask Wearing on ARI Incidence

**Figure 2:**
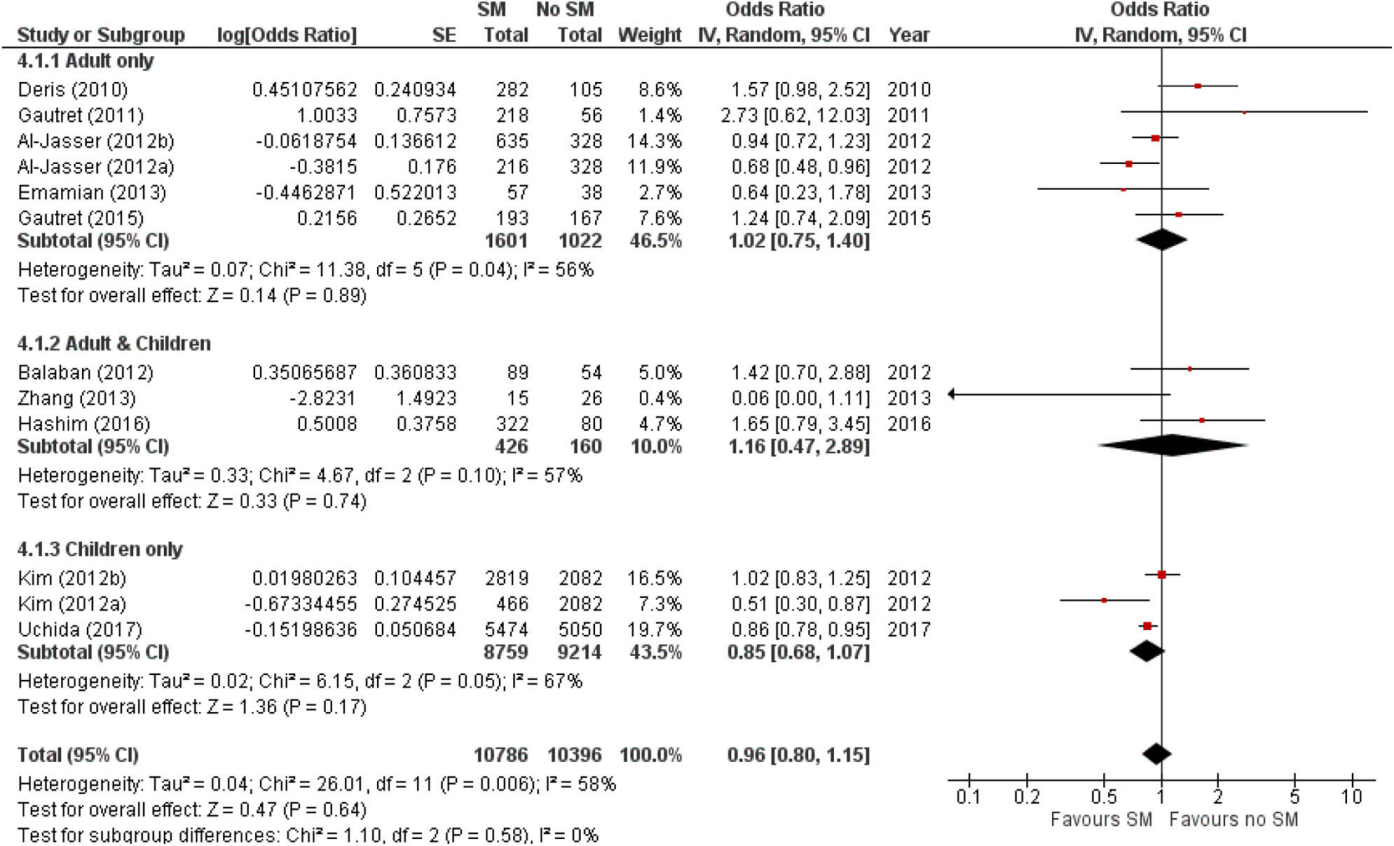
Pooled odds ratio for ARI incidence when surgical masks were worn, compared with not wearing surgical masks, in observational studies (n = 10 studies) estimated with the generic inverse method and a random-effects model. SM, surgical face mask; No SM, no surgical face mask; OR, odds ratio.

The estimated pooled odds ratio suggests that SM usage is not associated to preventing ARI incidence, and hence ineffective in preventing ARI incidence in non-healthcare settings. This is because the protective effect of SMs did not reach statistical significance (95% CI 0.8–1.15), although it lowered odds of ARI incidence by 4% compared with non-usage (pooled OR 0.96, Figure 2). Nonetheless, moderate heterogeneity was detected across the pooled studies (I^2^ = 58%, *p* = 0.006; Figure 2), indicating certain inconsistency in the findings on efficacy of SMs in ARI prevention.

**Figure 3:**
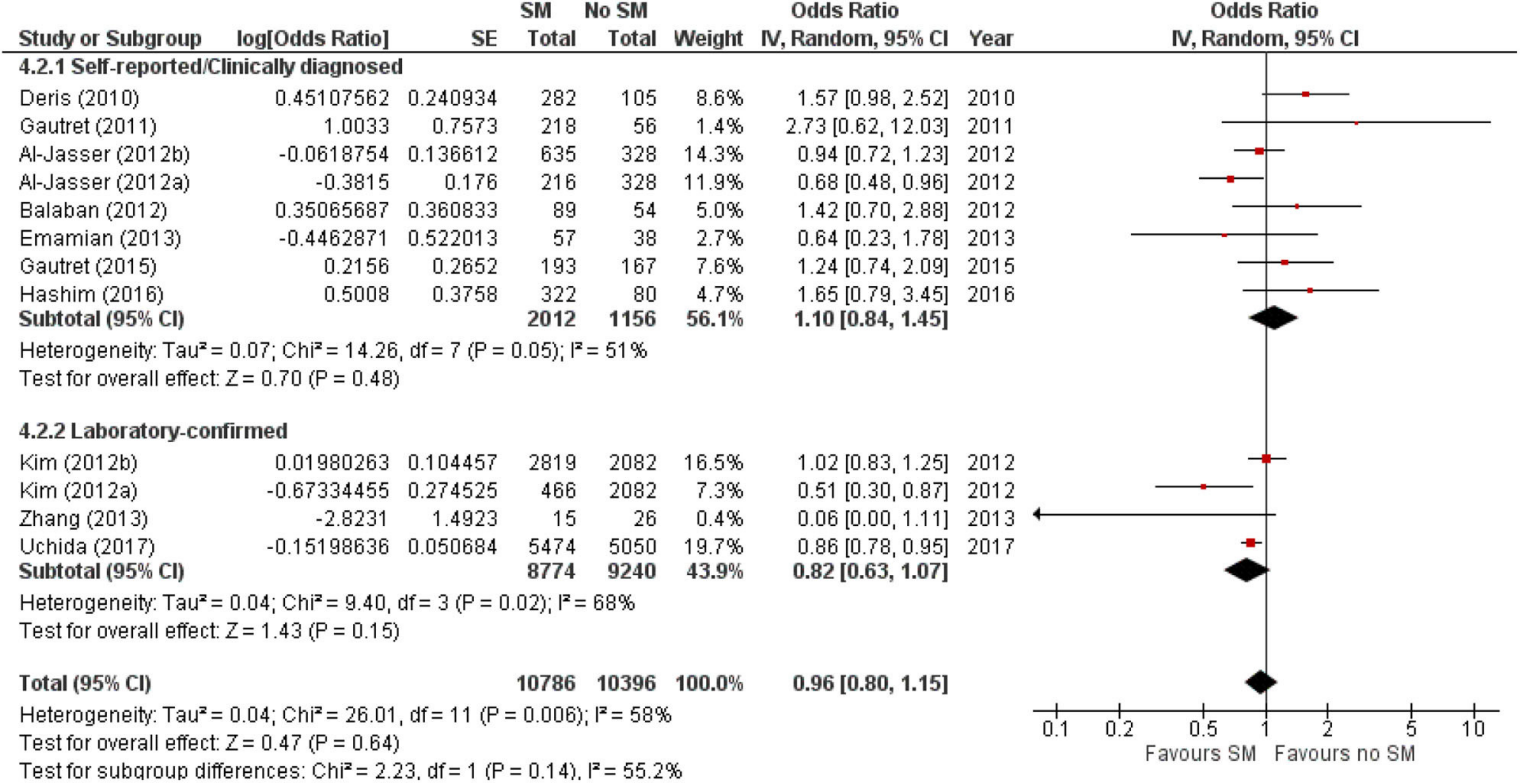
Subgroup analysis of ARI incidence when surgical masks were worn compared with not wearing surgical masks, according to outcome ascertainment methods, estimated with the generic inverse method and a random-effects model. SM, surgical face mask; No SM, no surgical face mask; OR, odds ratio.

The protective effect of SMs was more evident among children, demonstrated by a 15% lowered odds of ARI incidence (pooled OR 0.85; Figure 2). In contrast, increased odds for ARI incidence were observed among the adult-and-child and the adult-only populations with SM usage. SM usage was estimated to increase odds for ARI incidence in the adult-and-child population by 16% (pooled OR 1.16; Figure 2) and by 2% increased odds in the adult-only population (pooled OR 1.02; Figure 2). Nonetheless, the associations in all mentioned sub-populations were non-significant (95% CI: children-only: 0.75–1.40, adult-and-children: 0.4–2.89, adult-only: 0.75–1.40; Figure 2), indicating the ARI incidence was not associated with increased harm or protection from SM usage. Unexplained heterogeneity between studies were still observed in each subpopulation, particularly in the adult-only subpopulation (I^2^ = 56%, *p* = 0.04; Figure 2), with no statistically significant subgroup differences detected (*p* = 0.58; Figure 2). This suggests that age group of participants does not modify the effect of SM usage on ARI incidence, and hence is unlikely to be a factor behind the differential effects observed across pooled studies. Conventional and contoured funnel plots of the studies pooled in the meta-analysis suggests a slight asymmetry in the areas of mid to high statistical significance on the right side of the funnel plot (Supplementary Figures 1, 2). However, publication bias is unlikely to be the underlying cause of the observed plot asymmetry, as much as there is a lack of studies realizing a statistically significant harm associated with SM usage on ARI incidence. Subgroup analysis of the studies according to whether the ARI episode was laboratory-confirmed or not (i.e., self-reported or clinically confirmed) showed differential results on the effectiveness of SM usage on ARI prevention. A non-significant protective effect with SM usage was demonstrated when ARI incidence was laboratory-confirmed (pooled OR 0.82, 95% CI 0.63–1.07; Figure 3). When ARI incidence was self-reported or clinically confirmed, a non-significant harmful effect with SM usage was shown (pooled OR 1.10, 95% CI 0.84–1.45; Figure 3). Nonetheless, the subgroup difference detected was not statistically significant (*p* = 0.14; Figure 3). Unexplained inconsistencies in study findings was also detected within each subgroup, especially in the laboratory-confirmed outcomes subgroup where significant moderate heterogeneity was detected (I^2^ = 68%, *p* = 0.02). This indicates that the outcome ascertainment method used in pooled studies does not influence the association between SM usage and ARI incidence, and thus an unlikely cause for differential effects observed across pooled studies.

**Figure 4:**
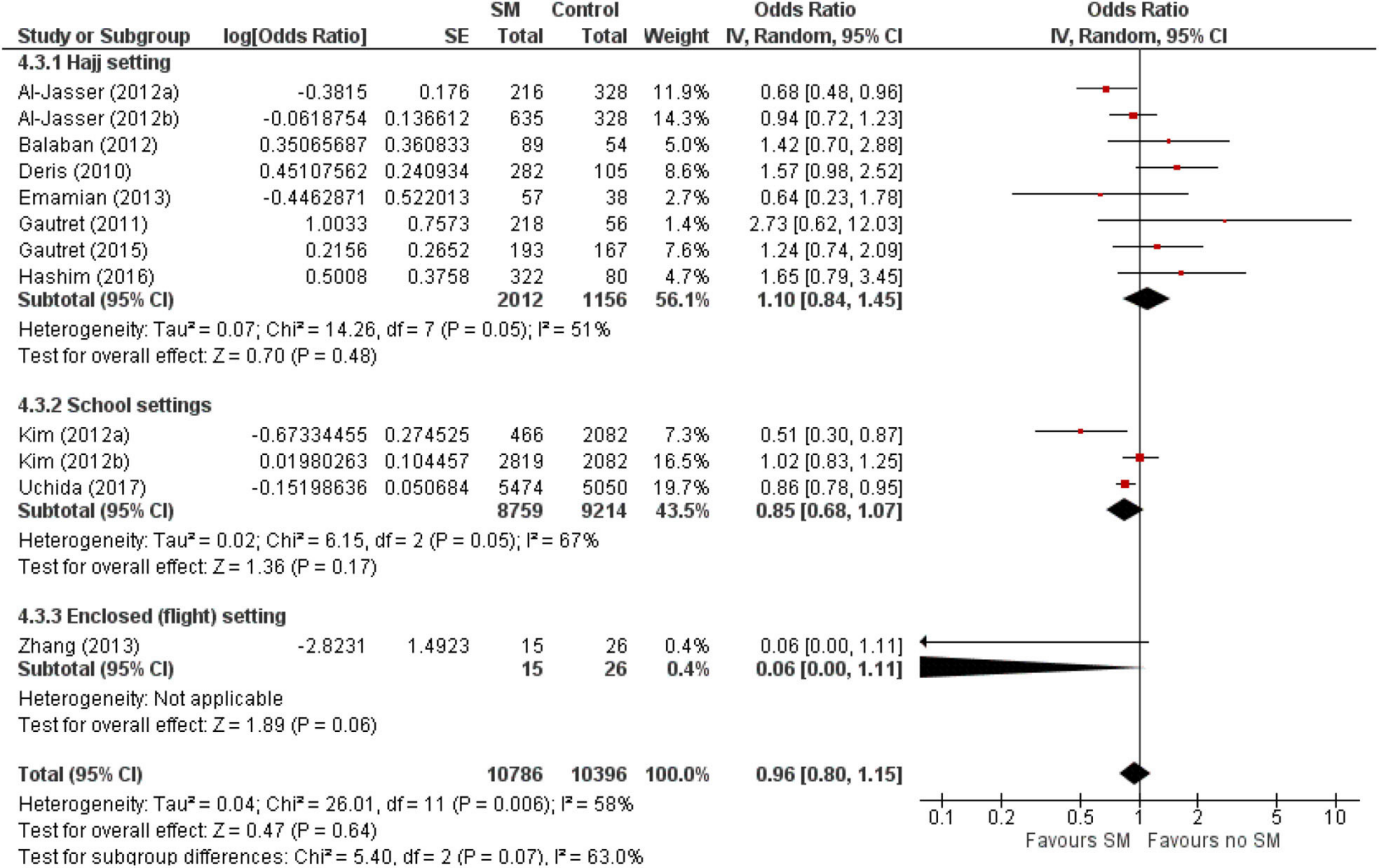
Pooled summary statistic for ARI incidence according to study settings, estimated with the generic inverse variance method and a random-effects model. SM, surgical face mask; No SM/HH, no surgical face mask or hand hygiene practices; OR, odds ratio.

When studies were stratified by study settings, results suggest that SM use has limited protective effect, and may even be harmful in mass gathering settings such as Hajj (pooled OR 1.10, 95% CI 0.45–1.45; Figure 4). However, in enclosed settings such as in schools or flights, a statistically non-significant protective effect against ARI was observed with SM use in studies conducted in schools, the same as that observed amongst children-only studies (Figure 4). This was attributable to the exact same studies included in these two subgroups.

Subgroup analysis of the outcome according to study design could not be performed in this review as there were insufficient interventional studies with suitable data to generate a pooled summary estimate.

**Figure 5:**
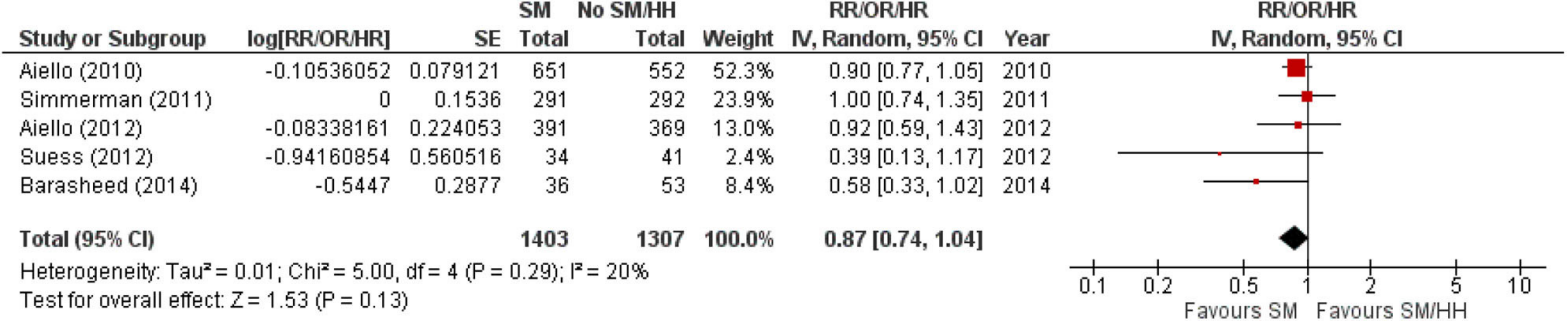
Pooled summary statistic for ARI incidence when surgical masks were worn compared with not wearing surgical masks in cluster randomized controlled trials, estimated with the generic inverse variance method and a random-effects model. SM, surgical face mask; No SM/HH, no surgical face mask or hand-hygiene practices; RR, risk ratio; OR, odds ratio; HR, hazard ratio.

However, the authors did estimate the pooled summary statistic on the effects of SM wearing on ARI incidence in cluster RCTs and found a similar non-significant protective effect of SM usage on ARI incidence (Figure 5). A 13% reduction in ARI incidence was noted with SM usage, compared with non-SM usage or implementation of hand-hygiene practices, although this reduction is not statistically significant (pooled summary statistic: 0.87, 95% CI 0.74–1.04; Figure 4). Nonetheless, the authors would like to highlight that the estimated pooled summary statistic only intends to provide a general idea on the direction of relationship between SM usage and ARI incidence. The pooled summary statistic in Figure 4 does not intend to, and is unable to quantitatively summaries the effects of SM usage on ARI incidence across the cluster RCTs included in this review. This is largely a result of the inaccuracy arising from reasons mentioned in Section Screening results and Characteristics of included studies.

### Reporting and Methodological Quality Assessment

High overall reporting quality was generally observed in the cluster RCTs, whereas only half of the observational studies had high overall reporting quality (25–27, 29, 30). The remaining observational studies had a low overall reporting quality, of which low-quality reporting was found in methods and results section of a study (31), and in either of the section in the remaining four studies (23, 24, 28, 31, 32) (Tables 3, 4).

**Table 4:**
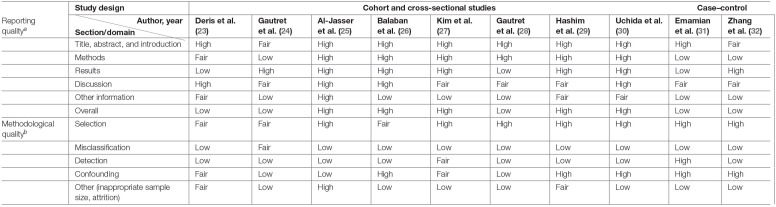
Summary of reporting and methodological quality of observational studies.

a *Reporting quality assessed with the STrengthening the Reporting of Observational Studies in Epidemiology (STROBE) statement*.

b *Methodological quality assessed with the National Heart, Lung and Blood Institute (NHLBI) Quality Assessment Tool for Observational Cohort, Cross-sectional, and Case–Control Studies*.

Methodological quality was poor in general across all studies. All cluster RCTs are at a high risk of overall bias, indicating poor overall methodological quality (Tables 3, 4). The generally high overall risk can be attributed to a high risk of bias from randomization as subversion was likely absent in all cluster RCTs. Bias from timing of identification and recruitment of individual participants in relation to timing of randomization is also likely to be present in two studies with baseline imbalances across groups that suggest the likelihood of recruitment bias (19, 21). Suess et al. (18) was at high risk of bias from reported deviations that arose because of trial context that may have affected the outcome. Three studies also measured multiple outcomes but reported only a single outcome (22), or measured the same outcome at multiple instances but only reported the outcome at a single instance (19, 21), rendering them at high risk of bias from selective reporting. The observational studies were generally at low risk of selection bias, except for three studies which were at moderate risk of selection bias (23, 24, 26). However, unclear or low participation rate was a common issue identified in all cross-sectional and cohort studies included in this review. All but three studies (23, 27, 29) did not specify the number of eligible subjects identified in the course of the study, of which two studies had a participation rate <50% (27, 29). Most studies were also at high risk of misclassification and detection bias, with only three studies at low or moderate risk of bias in these two domains (24, 27, 31). In the misclassification bias domain, a moderate- to high-risk recall bias mainly exists due to the use of retrospectively collected participant-reported exposures to assess exposure across all studies. It was also unclear whether a study utilizing a self-administered questionnaire was able to assess exposures across participants consistently because interpretation may vary across participants (23). Most of the observational studies were also at high risk of other biases, mainly arising from lack of sample size justification and/or attrition rates >20%.

Detailed results for the quality assessment of included studies can be found in Supplementary Tables 5–7.

## Discussion

### Effectiveness of Surgical Mask Usage on ARI Incidence in Non-healthcare Setting

Our results found that SM usage had a non-significant protective effect in reducing the risk of ARI among asymptomatic individuals in non-healthcare settings (pooled OR 0.96, 95% CI 0.8–1.15; Figure 2). The protective effect is also observed within or outside healthcare settings [healthcare setting pooled OR = 0.53; 95% CI 0.16–1.71 (17); community pooled RR = 0.78, 95% CI 0.51–1.20 (40)], regardless of those who were infected or uninfected (15). This contrasts with a review by Jefferson et al. (16), which found face mask to be the best performing intervention compared with other physical non-pharmaceutical interventions studied across different population and settings. Nonetheless, SM was only found to be significantly protective against SARS in the case–control subgroup (pooled OR 0.32, 95% CI 0.26–0.39), and studies related to SM usage was largely based in the healthcare setting and among healthcare workers (16). This limits the generalizability of the study given the different compliance in SM wearing between healthcare workers and the general population.

This review observed a non-significant protective effect that was more prominent in the younger age group (pooled OR 0.85, 95% CI 0.75–1.40; Figure 2). This contradicts with an experimental study that assessed transmission reduction potential by personal respirators, surgical masks, and homemade masks. The study attributed a significantly less protective effect of all types of mask usage for children, plausibly due to an inferior fit of masks on their smaller faces (41). The observed age-specific difference in effect is also likely because majority of the observational studies among the adult and adult-and-children populations were conducted in Hajj settings. As shown in Figure 4, there were differential effects of SM use in mass gatherings such as Hajj, and other enclosed settings in schools or flights. The annual Hajj, which involves as many as 2 million pilgrims nested in highly dense areas for a prolonged period, cannot be generalized to a regular community. Other conditions favoring the spread of infectious diseases include the physical exertion of pilgrims in overcrowded conditions, limited access to resources, humid conditions, and low compliance to mask usage due to religious beliefs (42–44). The combined effects of greater compliance with mask usage and hygiene practices in a more controlled environment could amplify the protective effect of SM usage among children.

SM use was protective against ARI incidence when outcomes were laboratory-confirmed episodes (pooled OR 0.82, 95% CI 0.63–1.07; Figure 3), but harmful when outcomes were self-reported or clinically diagnosed. The contrasting observations might be attributed to the subjective nature of self-reported or clinically diagnosed outcome and retrospective collection of self-reported outcomes in most included studies. Such data collection methods are liable to inaccuracies from the participants' judgment of personal condition and recall bias (45). Effects of SM usage in studies utilizing self-reported or clinically diagnosed episodes could also have been diminished by (1) participants overstating the actual experience of illness at the baseline or understating the condition at the end point, and (2) the inability to detect asymptomatic carriers. Conversely, a laboratory-confirmed outcome is more objective and does not require any participant judgment, enabling more accurate evaluation of ARI even among asymptomatic participants.

The effectiveness of face mask in source control hinges on the specific mode of transmission of etiological agent. Studies included in this review measured influenza or ILI, which are collectively caused by a broad range of viruses of varying infectivity and transmission routes (46). Direct and indirect contact are the primary transmission routes of respiratory syncytial virus and adenovirus, which causes ILI, whereas SARS is mainly spread through contact and droplet transmission (3, 47). Influenza is thought to be primarily transmitted through droplet expulsion, although evidence supporting airborne transmission is growing (48). A recent study found significant reduction in influenza virus emitted through droplets and not aerosols produced by infected individuals after SM usage (13). Another study showed that SM is more effective at reducing influenza viral RNA copies in coarse particles >5 μm (25-fold) than fine aerosols <5 μm (2.8-fold) emitted by an infected wearer (12). SM's effectiveness in preventing influenza decreases with decreasing particle size. As short-range aerosol inhalation is currently the main transmission mode of SARS-CoV-2 in the ongoing COVID-19 pandemic, the usage of SMs may not be highly effective to filter these fine aerosols completely.

The effectiveness of SM usage at reducing environmental risk faced by uninfected individuals remains unclear as existing evidence is limited to mechanistic challenge on masks with largely conflicting results. A study observed lower amount of influenza virus by 1.1- to 55-fold with an average of 6-fold with varying SM design (49). Conversely, SM has also been found to allow penetration of particles as small as 0.04–0.2 μm (influenza virus: 0.08–0.12 μm) (50). Specifically, Bae et al. (51) reported a low effectiveness of filtering SARS-CoV-2 on the basis of the small particle size as SARS-CoV (0.08–0.14 μm). At the time of conducting this review, research found increasing evidence of asymptomatic transmission of SARS-CoV-2 (10, 11). A sweeping change in recommendations to encourage SM usage by the general public was made amidst growing concerns of an increasing asymptomatic infected population, to prevent asymptomatic infected individuals from exposing uninfected individuals to the virus. Despite limited evidence on SM's effectiveness in reducing SARS-CoV-2 transmission due to its plausible airborne transmission mode and small viral particle size, the mechanistic feasibility of masking combined with large-scale uptake by populations might reap effectiveness that have yet to be measured in clinical trials (52).

### Effectiveness of Hand Hygiene on ARI Incidence

Three studies included in this review also found no significant protective effect of SM coupled with hand-sanitizer use (SM+HH) on ARI incidence (18, 19, 21) compared with control groups [adjusted HR (95% CI) 0.87 (0.73–1.02) (19), 0.78 (0.57–1.08) (21), adjusted OR (95% CI) 0.62 (0.23–1.65) (18); Appendix D, Supplementary Table 8]. However, the results could have been limited by differential protective effect conferred by different types of hand sanitizer used and potential improper application. Gel-based sanitizers were used in two studies (19, 21) and the remaining study likely used liquid-based sanitizer (18). The superiority of liquid-based hand sanitizers to its gel-based counterparts may have resulted in the lack of effectiveness observed (53). More recent evidence also points to increased effectiveness of hand sanitizer in reducing microorganism burden when properly applied in accordance to EN 1500 standards (54).

Nonetheless, WHO recommends that masks are only effective when used in tandem with proper and frequent hand hygiene (55). Findings from a household cluster RCT (56) suggested the risk of influenza transmission is significantly low when healthy family members practice SM usage and frequent hand hygiene within 36 h of symptom onset of an infected family member [adjusted OR 0.16, 95% (CI 0.03–0.92), *p* = 0.04 (18)]. There is also evidence on hand washing with soap and/or hand sanitizer's effectiveness in removing influenza virus (57, 58). These conflicted findings supported inconclusive findings from a review on hand hygiene's protective effect in the community setting (59). Hence, although hand hygiene shows potential in reducing influenza infection and transmission, its effectiveness depends on the types of hand hygiene practiced (e.g., hand sanitizer, washing with soap, and water), usage frequency, proper application, and the setting in which practices are implemented.

Apart from use of SM and hand hygiene, other non-pharmaceutical interventions commonly employed in conjunction will also influence the risk of ARI in the community (60). However, the effectiveness of these measures is beyond the scope of this review and has been extensively evaluated in this other recently published review (61).

### Strengths and Limitations

A strength of this review lies in the comprehensive outcomes examined, utilizing an extensive list of pathogens referenced from Jefferson et al. (16) in our search strategy. This compares with the review by Xiao et al. (40) which examined laboratory-confirmed influenza outcomes, or that by Cowling et al. (15) which focused on influenza, flu, and respiratory infections. The period in which evidence for this review was collected also sets it apart from existing reviews. Jefferson et al. (16) explored studies from 1980 to 2010 and Xiao et al. (40) went as far back as 1946. By including studies published in the past decade only, this review presents evidence more representative of the current and rapidly evolving environmental and social–behavioral factors. Next, no limits were imposed on health status and age of populations studied. This diversification increases the representativeness and generalizability of our findings to a community profile. Collectively, the study constitutes an update of SM efficacy investigated in Jefferson's review (16).

This review also complements the review by Xiao et al. (40) in terms of intervention and population evaluated. We focused on assessing SM usage, a more feasible apparatus for the general public as opposed to more intricate facial protective gear included in Xiao et al. (40), that should be reserved for healthcare workers or vulnerable populations. Unlike Xiao et al. (40), this review focused solely on assessing SM's efficacy among the uninfected population. In addition, the included studies were assessed for reporting and methodological qualities, providing additional insight to the reported findings and inadequacy in existing studies to constitute strong evidence on SM efficacy.

However, limitations exist in this review. First, the study settings were largely homogenous. More than half of the studies included were conducted during Hajj, which is unique and distinctly different from the general community setting. This limits the external validity of our results. Second, the authors pooled adjusted summary measures of studies included in the meta-analysis, but residual confounding may still exist in the reported summary measures. Third, the RCTs included cannot be pooled accurately as summary estimates reported were incompatible for a meta-analysis. Analysis of pooled RCT data, where SM use is better complied with and purposely differentiated between groups, would have constituted more robust evidence on the efficacy of SMs between wearers and non-wearers. Fourth, poor methodological quality was determined across all studies included in this analysis. This review highlights a paucity in well-conducted research examining the efficacy/effectiveness of SMs against ARI incidence in the general community. Non-standardization of methodologies and assessed outcomes inhibited accounting for inconsistencies in compliance to SM usage by the study populations, likely undermining the effectiveness of SMs in preventing ARI. On the contrary, compliance tend to be unusually high during epidemics due to increased risk perception (62). Next, although the funnel plots only showed slight asymmetry (Supplementary Figures 1, 2), there is a likelihood of publication bias as we only searched published literature. Lastly, there is diminished relevance for SM use in resource constrained conditions amidst epidemics. The strict focus on SM in this review was to examine the effectiveness of personal protection equipment that was accessible for the general population. However, there is extensive substitution of SM using reusable cloth masks or face coverings in the current pandemic due to supply constraints. Their efficacy/effectiveness in preventing ARI have not been widely evaluated, and future trials should compare the efficacy/effectiveness of reusable cloth masks to a standard (either SMs or even respirators) to inform policies on cloth mask usage. Nonetheless, such trial findings need to be interpreted with caution as cloth mask production is not regulated, and the quality and construct between products in this category may vary widely. This is unlike SMs which have a consistent quality and construct due to them being a regulated product under 21 CFR 878.4040 (63).

Existing studies are characterized by weak methodologies and a lack of overall significant effect, possibly constrained by small sample sizes. Well-designed, executed, and adequately funded trials are needed to provide robust evidence on SM efficacy in reducing transmission in the general community. Larger studies could be beneficial given their increased sensitivity to small effect sizes, and RCTs have the added benefit of establishing causality over observational studies (although it may be more resource intensive). Well-designed *in vivo* studies on uninfected individuals wearing SM could also be conducted to investigate the proportion of virus of varying sizes that can be blocked by SM via droplet or aerosolized transmission. These studies would form the basis of strong evidence to inform policies and practices on SM usage in the community during times of pandemic.

## Conclusion

Our review found that SMs were not associated to ARI incidence, indicating that SMs may be ineffective in preventing respiratory illness when worn by an uninfected individual in the general community. However, given the weak methodologies across studies assessed and the possibility of residual confounding, an absence of evidence cannot be simply regarded as an evidence of absence. SM usage cannot be a standalone strategy to protect against infection, but ought to be used together with other physical intervention methods such as hand hygiene and social distancing to combat multiple modes of virus transmission in the community.

## Data Availability Statement

All datasets generated for this study are included in the article/Supplementary Material.

## Author Contributions

JP conceptualized the study. MW, SG, and PC drafted the article, screened the studies, and extracted data. MW analyzed data and prepared the figures and tables. JP validated the final screened studies and analysis, critically edited draft, and interpretation of the findings. All authors have read and agreed to the published version of the article.

## Conflict of Interest

The authors declare that the research was conducted in the absence of any commercial or financial relationships that could be construed as a potential conflict of interest.
