# Supplementary material for: Effectiveness of Surgical Face Masks in Reducing Acute Respiratory Infections in Non-Healthcare Settings: A Systematic Review and Meta-Analysis
Source: Front Med (Lausanne). 2020 Sep 25;7:564280. doi: 10.3389/fmed.2020.564280 (PMC7546829; doi:10.3389/fmed.2020.564280)
Supplement: Supplementary file 1 [file Data_Sheet_1.docx]

# Supplementary Material

### Table S1. Description of PICOS/PEOS criteria for a systematic review assessing the association of micronutrient deficiency with the incidence, duration and severity of common cold and pneumonia.

| **Variable** | **Description** |
| --- | --- |
| Population | Humans in the general community setting, not healthcare workers or humans in clinical and medical setting |
| Intervention/Exposure | Wearing of surgical face masks only |
| Comparator | A: No surgical face masks worn  B: Hand hygiene practices |
| Outcome | Incidence or episodes of i) acute respiratory infectious disease and ii) non-influenza respiratory outbreaks in a community setting |
| Study Design | Randomised controlled trial and Observational studies (except for case reports) |
| Research Question | Does wearing a surgical mask decrease incidence or is associated to decreased incidence of i) respiratory infectious disease and ii) non-influenza respiratory outbreaks in humans in a community setting, compared to not wearing a surgical mask or having only hand hygiene practices? |

### Table S2. Search strategy utilised in Search strategy for micronutrient deficiency of cold and/or pneumonia PubMED, Embase and the Cochrane Library

| **Database** | **Search terms** | **PICOS/PEOS criteria** | **Filters applied** | **Results yielded** |
| --- | --- | --- | --- | --- |
| PubMED | NOT (((((((((nosocomial infection) OR (nosocomial infection[MeSH Terms])) OR (healthcare workers)) OR (health care workers)) OR (healthcare personnel)) OR (health care personnel))) OR (in-vitro study[Title/Abstract])) OR (animal[Title/Abstract])) | Population | Publication Year: 2010 - 2020 |  |
|  | ((prevent*) OR (prevention and control[MeSH Subheading]))) AND ((face mask) OR (surgical mask) OR (facemask) OR (mask*))) | Intervention/ Exposure |  |  |
|  | (((((((((((((((((((((((((((((((((((((((flu) OR (influenza)) OR (influenza-like illness)) OR (respiratory virus)) OR (respiratory infection)) OR (respiratory tract infection)) OR (respiratory infectious disease)) OR (severe acute respiratory syndrome)) OR (SARS)) OR (middle east respiratory syndrome)) OR (MERS)) OR (severe acute respiratory syndrome[MeSH Terms]))) OR (rhinovirus*)) OR (adenovirus*)) OR (adenoviridae infections, human[MeSH Terms])) OR (coronavirus[MeSH Terms])) OR (coronavir*)) OR (respiratory syncytial virus*)) OR (Respiratory Syncytial Viruses, Human[MeSH Terms])) OR (Parainfluenza Virus 1, Human[MeSH Terms])) OR (Parainfluenza Virus 2, Human[MeSH Terms])) OR (Parainfluenza Virus 3, Human[MeSH Terms])) OR (Parainfluenza Virus 4, Human[MeSH Terms])) OR ("para?influenza virus")) OR (enterovirus infection[MeSH Terms])) OR (enterovir*)) OR (bocavir*)) OR (human bocavirus[MeSH Terms])) OR (human metapneumovirus[MeSH Terms])) OR (metapneumovir*)) OR (parvovir* infection)) OR (erythema infectiosum)) OR (parvovirus b19, human[MeSH Terms])) OR (human parechovirus[MeSH Terms])) OR (parechovir*)) NOT (chronic)) | Outcome |  |  |
|  | - | Study Design |  | 82 |
|  | PICOS/PEOS | |  | 336 |
| Embase | 'human'/exp NOT ('health care personnel'/exp OR 'health care personnel' OR 'health care practitioner' OR 'health care professional' OR 'health care provider' OR 'health care worker' OR 'health personnel' OR 'health profession personnel' OR 'health worker' OR 'healthcare personnel' OR 'healthcare practitioner' OR 'healthcare professional' OR 'healthcare provider' OR 'healthcare worker' OR 'home health aides' OR 'personnel, health' OR 'public health officer') | Population | Publication Year: 2010 - 2020 | 0 |
|  | ('face mask'/exp OR 'surgical mask'/exp OR 'barrier (device)' OR 'aseptex surgical mask' OR 'mask, surgical' OR 'surgery mask' OR 'surgical face mask' OR 'surgical face mask (physical object)' OR 'surgical face mask, reusable' OR 'surgical face mask, single-use' OR 'surgical mask' OR 'surgical masks') AND 'prevention and control'/exp | Intervention/ Exposure |  |  |
|  | ('influenza'/exp OR 'bronchitis epidemica' OR 'bronchitis, epidemic' OR 'epidemic bronchitis' OR 'flu' OR 'flue' OR 'influenza' OR 'influenza infection' OR 'influenza syndrome' OR 'influenza, human' OR 'common cold'/exp OR 'cold, common' OR 'common cold' OR 'common colds' OR 'coryza' OR 'natural cold' OR 'natural colds' OR 'human rhinovirus'/exp OR 'human rhinovirus' OR 'common cold virus' OR 'rhinovirus, human' OR 'virus, common cold' OR 'adenoviridae'/exp OR 'adenoviridae' OR 'adeno virus' OR 'adenovirus' OR 'virus, adeno' OR 'human respiratory syncytial virus'/exp OR 'human orthopneumovirus' OR 'human respiratory syncytial virus' OR 'rs virus (lung)' OR 'rs virus (pulmonary)' OR 'rsv (lung)' OR 'respiratory syncytial pneumovirus' OR 'respiratory syncytial virus' OR 'syncytial virus' OR 'chimpanzee coryza agent' OR 'respiratory chimpanzee coryza agent' OR 'respiratory syncitial virus' OR 'respiratory syncytial virus, human' OR 'respirosyncytial virus' OR 'syncytial respiratory virus' OR 'virus, respiratory syncytial' OR 'pneumovirus infection'/exp OR 'pneumovirus infection' OR 'pneumovirus infections' OR 'human parainfluenza virus 1'/exp OR 'human parainfluenza virus 1' OR 'parainfluenza 1' OR 'parainfluenza virus 1' OR 'hemadsorption virus 1' OR 'hemagglutinating japan virus' OR 'hvj virus' OR 'influenza d virus' OR 'newborn pneumonitis virus' OR 'para influenza virus 1' OR 'para-influenza virus type 1' OR 'parainfluenza 1 virus' OR 'parainfluenza virus 1, human' OR 'parainfluenza virus type 1' OR 'sendai paramyxovirus' OR 'human parainfluenza virus 2'/exp OR 'human parainfluenza virus 2' OR 'human rubulavirus 2' OR 'parainfluenza 2' OR 'parainfluenza virus 2' OR 'para influenza virus 2' OR 'para-influenza virus type 2' OR 'parainfluenza 2 virus' OR 'parainfluenza virus 2, human' OR 'parainfluenza virus type 2' OR 'human parainfluenza virus 3'/exp OR 'human parainfluenza virus 3' OR 'parainfluenza 3' OR 'parainfluenza virus 3' OR 'myxovirus parainfluenza 3' OR 'para influenza 3' OR 'para influenza virus 3' OR 'para-influenza virus type 3' OR 'parainfluenza 3 virus' OR 'parainfluenza virus 3, human' OR 'parainfluenza virus type 3' OR 'human parainfluenza virus 4'/exp OR 'human parainfluenza virus 4' OR 'human rubulavirus 4' OR 'parainfluenza 4' OR 'parainfluenza virus 4' OR 'parainfluenza virus 4, human' OR 'parainfluenza virus type 4' OR 'virus, parainfluenza type 4' OR 'enterovirus infection'/exp OR 'enterovirus infection' OR 'enterovirus infections' OR 'enteroviral infection' OR 'infection, enterovirus' OR 'human bocavirus'/exp OR 'human bocavirus' OR 'metapneumovirus infection'/exp OR 'metapneumovirus infection' OR 'human parvovirus b19'/exp OR 'human parvovirus b19' OR 'parvovirus b19' OR 'parvovirus b19, human' OR 'parvoviridae'/exp OR 'parvoviridae' OR 'parvo virus' OR 'parvovirus' OR 'parvovirus, feline' OR 'pico virus' OR 'picodna virus' OR 'picodua virus' OR 'human parechovirus'/exp OR 'human parechovirus' OR 'parechovirus a' OR 'respiratory tract infection'/exp OR 'acute respiratory infection' OR 'acute respiratory tract infection' OR 'airway infection' OR 'bronchopulmonary infection' OR 'chronic respiratory tract infection' OR 'infection, respiratory tract' OR 'pulmonary tract infection' OR 'respiration infection' OR 'respiration tract infection' OR 'respiratory infection' OR 'respiratory tract infection' OR 'respiratory tract infections' OR 'coronavirus infection'/exp OR 'coronavirus infection' OR 'coronavirus infections' OR 'severe acute respiratory syndrome'/exp OR 'sars' OR 'severe acute respiratory syndrome' OR 'sudden acute respiratory syndrome' OR 'sars-related coronavirus'/exp OR 'sars-like cov' OR 'sars-like coronavirus' OR 'sars-related cov' OR 'sars-related coronavirus' OR 'sarsr-cov' OR 'severe acute respiratory syndrome-like coronavirus' OR 'severe acute respiratory syndrome-related coronavirus' OR 'middle east respiratory syndrome'/exp OR 'middle east respiratory syndrome' OR 'middle east respiratory syndrome coronavirus infection') | Outcome |  |  |
|  | - | Study Design |  |  |
|  | PEOS | |  | 621 |

| **Database** | **Search ID** | **Search terms** | **PICOS/PEOS criteria** | **Filters Applied** | **Results yielded** |
| --- | --- | --- | --- | --- | --- |
| Cochrane Library | #1 | MeSH descriptor: [Influenza, Human] explode all trees | Outcome |  |  |
|  | #2 | influenza:ti,ab,kw |  |  |  |
|  | #3 | flu:ti,ab,kw |  |  |  |
|  | #4 | MeSH descriptor: [Common Cold] explode all trees |  |  |  |
|  | #5 | common cold:ti,ab,kw |  |  |  |
|  | #6 | MeSH descriptor: [Rhinovirus] explode all trees |  |  |  |
|  | #7 | rhinovirus*:ti,ab,kw |  |  |  |
|  | #8 | MeSH descriptor: [Adenoviridae Infections] explode all trees |  |  |  |
|  | #9 | MeSH descriptor: [Adenoviridae] explode all trees |  |  |  |
|  | #10 | adenovirus*:ti,ab,kw |  |  |  |
|  | #11 | MeSH descriptor: [Coronavirus] explode all trees |  |  |  |
|  | #12 | MeSH descriptor: [Coronavirus Infections] explode all trees |  |  |  |
|  | #13 | coronavirus*:ti,ab,kw |  |  |  |
|  | #14 | MeSH descriptor: [Respiratory Syncytial Viruses] explode all trees |  |  |  |
|  | #15 | MeSH descriptor: [Respiratory Syncytial Virus Infections] explode all trees |  |  |  |
|  | #16 | respiratory syncytial virus*:ti,ab,kw |  |  |  |
|  | #17 | respiratory syncythial virus:ti,ab,kw |  |  |  |
|  | #18 | MeSH descriptor: [Parainfluenza Virus 1, Human] explode all trees |  |  |  |
|  | #19 | MeSH descriptor: [Parainfluenza Virus 2, Human] explode all trees |  |  |  |
|  | #20 | MeSH descriptor: [Parainfluenza Virus 3, Human] explode all trees |  |  |  |
|  | #21 | MeSH descriptor: [Parainfluenza Virus 4, Human] explode all trees |  |  |  |
|  | #22 | (parainfluenza OR para-influenza OR para influenza):ti,ab,kw |  |  |  |
|  | #23 | MeSH descriptor: [Severe Acute Respiratory Syndrome] explode all trees |  |  |  |
|  | #24 | MeSH descriptor: [SARS Virus] explode all trees |  |  |  |
|  | #25 | (SARS OR severe acute respiratory syndrome):ti,ab,kw |  |  |  |
|  | #26 | MeSH descriptor: [Middle East Respiratory Syndrome Coronavirus] explode all trees |  |  |  |
|  | #27 | (MERS OR Middle East Respiratory Syndrome):ti,ab,kw |  |  |  |
|  | #28 | MeSH descriptor: [Respiratory Tract Infections] explode all trees |  |  |  |
|  | #29 | acute respiratory tract infection*:ti,ab,kw |  |  |  |
|  | #30 | acute respiratory infection*:ti,ab,kw |  |  |  |
|  | #31 | MeSH descriptor: [Enterovirus Infections] explode all trees |  |  |  |
|  | #32 | enterovir*:ti,ab,kw |  |  |  |
|  | #33 | MeSH descriptor: [Human bocavirus] explode all trees |  |  |  |
|  | #34 | bocavirus*:ti,ab,kw |  |  |  |
|  | #35 | MeSH descriptor: [Metapneumovirus] explode all trees |  |  |  |
|  | #36 | metapneumovir*:ti,ab,kw |  |  |  |
|  | #37 | MeSH descriptor: [Parvovirus B19, Human] explode all trees |  |  |  |
|  | #38 | (parvoviridae infections OR erythema infectiosum):ti,ab,kw |  |  |  |
|  | #39 | parvovirus*:ti,ab,kw |  |  |  |
|  | #40 | MeSH descriptor: [Parechovirus] explode all trees |  |  |  |
|  | #41 | parechovirus*:ti,ab,kw |  |  |  |
|  | #42 | {OR #1-#41} |  |  |  |
|  | #43 | MeSH descriptor: [Public Health Practice] explode all trees |  |  |  |
|  | #44 | Preven*:ti,ab,kw |  |  |  |
|  | #45 | MeSH descriptor: [Masks] explode all trees | Intervention/ Exposure |  |  |
|  | #46 | Mask*:ti,ab,kw |  |  |  |
|  | #47 | #45 or #46 |  |  |  |
|  | #49 | MeSH descriptor: [Health Personnel] explode all trees | Population |  |  |
|  | #50 | (healthcare workers or healthcare personnel):ti,ab,kw |  |  |  |
|  | #51 | #48 not (#49 or #50) in Cochrane Reviews, Trials | PICOS/PEOS | Custom year range from Jan 2010- 25 Feb 2020 | 126 |

# Appendix B.

## Methodological and Reporting Quality Assessment methodology and detailed results

### Methods for methodological quality assessment

The methodological quality was assessed with the National Lung, Heart, and Blood Institute (NLHBI) Quality Assessment Tool for Observational Cohort, Cross-sectional and Case-Control Studies [5]. The tool was used to appraise the risk of bias in the domains of selection, misclassification, detection, confounding, attrition and inappropriate sample sizes for each study. Each question was answered using the guideline mentioned in the website, which did not mention an approach to summarise the methodological qualities of studies. Thus, the review authors grouped questions into the identified bias domains (Table B1, colour coded in Table B2) and assigned the domain-specific quality using the majority of the responses to questions in that domain. A majority of “yes”, “can’t tell/partially” and “no” responses for questions in the domain corresponded to high, fair and low quality for the domain. In the case of a split response i.e. equal number of differing responses in the domain, the lower quality is assigned as the domain-specific quality for a conservative judgement.

The review authors did attempt to generate an overall methodological quality for each study by assigning the lowest quality given across all assessed domains as the overall methodological quality. This proposed approach assumed that all domains contributed equally to the overall quality, but also resulted in the unequal weighting of questions in the tool. This is especially so for domains with only one question assessing the risk of bias in that domain e.g. other bias and confounding domains. This approach may then be flawed as the overall methodological quality could potentially be dependent on the response to a single question. Thus, the proposed approach to summarise the overall methodological quality using the lowest domain-specific quality may not be robust as it could not convey the methodological quality of a study concisely but transparently. Therefore, the review authors did not generate an overall methodological quality for each study, but only reported the domain-specific qualities for each study to give a more accurate reflection of the methodological quality, and hence the risk of biases for each study (Table B3).

### Table S3. Classification of risk of bias domains assessed with published questions in NLHBI quality assessment tool for observational cohort, cross-sectional and case-control studies.

| Domain of Bias Assessed | Cohort & Cross sectional Studies | Case-control studies |
| --- | --- | --- |
| Selection | Questions 2 - 4 | Questions 2, 4 - 8 |
| Misclassification | Questions 6 - 10 | Questions 9 - 10 |
| Detection | Questions 11, 12 | Question 11 |
| Confounding | Question 14 | Question 12 |
| Other (Inappropriate sample size, attrition) | Questions 5, 13 | Question 3 |

### Methods for reporting quality assessment

Reporting quality was assessed with the STrengthening the Reporting of OBservational studies in Epidemiology (STROBE) statement [6,7]. The statement was utilised as a checklist with “yes”, “no” and “partially” given as responses when questions were applicable to the given study design. “Yes” was given when the element raised by the question was reported in the study, “no” when the element is absent from the study and “partially” when the element was only partially reported in the study. The review authors then summarised the quality for each section using the majority of responses for that given section. A majority of “yes”, “partially” and “no” responses for questions in the section corresponded to high, fair and low quality for that given section. In the case of a split response i.e. equal number of differing responses in the section, the lower quality is assigned as the quality of the section for a conservative judgement. The review authors then used the quality of the methods, results and discussion sections to generate an overall reporting quality for the study, as these sections were deemed as the core sections in a publication. The title, abstract and introduction sections were not included in the framework to determine overall reporting quality as they do not significantly affect the overall reporting qualities of studies included, even when taken into consideration as shown in the results (Table B3). The proposed frameworks for assigning overall reporting quality based on the quality of the methods, results and discussion section of each study is shown in Table S4(A), and that including the title, abstract and introduction sections is shown in Table S4(B).

### **Table S4**. Proposed framework for determining overall reporting quality based on title, abstract and introduction, methods, results and discussion sections

| **Overall quality** | **Quality of section** | |
| --- | --- | --- |
| Sections considered in framework | Methods, Results and Discussion (A) | Title and abstract, Introduction, Methods, Results and Discussion (B) |
| High | All M, R, D = high | All I, M, R, D = high |
|  | 2 of M, R, D = high  1 of M, R, D = fair | 3 of I, M, R, D = high  1 of I, M, R, D = fair |
|  |  | 2 of I, M, R, D = high  2 of I, M, R, D = fair |
| Fair | All M, R, D = fair | All I, M, R, D = fair |
|  | 2 of M, R, D = high  1 of M, R, D = low | 3 of I, M, R, D = high  1 of I, M, R, D = low |
|  |  | 1 of I, M, R, D = high  3 of I, M, R, D = fair |
|  | 1 of M, R, D = high  2 of M, R, D = fair | 2 of I, M, R, D = high  2 of I, M, R, D = low |
|  |  | 2 of I, M, R, D = high  1 of I, M, R, D = fair  1 of I, M, R, D = low |
| Low | All M, R, D = low | 3 of I, M, R, D = fair  1 of I, M, R, D = low |
|  | 1 of M, R, D = high 1 of M, R, D = fair 1 of M, R, D = low | 1 of I, M, R, D = high  3 of I, M, R, D = low |
|  |  | 1 of I, M, R, D = high 2 of I, M, R, D = fair 1 of I, M, R, D = low |
|  | 1 of M, R, D = high/fair 2 of M, R, D = low | 1 of I, M, R, D = high 1 of I, M, R, D = fair  2 of M, R, D = low |
|  | 2 of M, R, D = fair 1 of M, R, D = low | ≤2 of I, M, R, D = fair ≥2 of I, M, R, D = low |

I, Title, Abstract and Introduction; M, Methods; R, Results; D, Discussion

### Table S5. Details of methodological quality assessment for each included randomised controlled trial using the Risk of Bias Assessment Tool 2.0 (RoB 2.0) for Cluster-randomised Trials

| General Information | First Author, Year | Aiello. 2012 | Simmerman, 2011 | Aiello, 2012 (1) | Aiello, 201 (2) | Suess, 2012 (1) | Suess, 2012 (2) | Barasheed, 2014 |
| --- | --- | --- | --- | --- | --- | --- | --- | --- |
|  | Study design | Cluster-randomised trials | | | | | | |
|  | Specify intervention vs control arm being assessed | Intervention: Mask Wearing in residential hall  Control: basic hand hygiene education | Intervention: Mask Wearing in household **+ hand washing** VS Control: Hand hygiene education + handwashing kit | Intervention: face mask and hand hygiene  Control: Basic education on proper hand hygiene and use of standard surgical face masks | | Intervention: Healthy household members to wear masks at all times when in one room with the index patient and/or any other household member with respiratory symptoms  Control: Provision of general information on infection control to household | | Intervention: Provision of face masks, and advice and instructions on mask usage through participants’ stay in Mina Control: No face masks provided; only general information on hygiene was provided |
|  | Specify outcome being assessed | Self-reported/ Clinically confirmed ILI | secondary influenza | laboratory-confirmed influenza A/B infection | self-reported ILI | laboratory confirmed influenza infection | Clinically confirmed ILI | Secondary influenza-like-illness |
|  | Specify the numerical result being assessed.  In case of multiple alternative analyses being presented, specify the numeric result (e.g. RR = 1.52 (95% CI 0.83 to 2.77) and/or a reference (e.g. to a table, figure or paragraph) that uniquely defines the result being assessed. | Adjusted HR: 0.9 (95% CI: 0.77-1.05); proportion of participants with ILI | proportion of participants with secondary influenza | Adjusted HR: 0.92 (95% CI: 0.59, 1.42), proportion of participants with laboratory confirmed influenza episode | Adjusted HR: 1.1 (95% CI 0.88-1.38); proportion of participants with ILI | Adjusted OR: 0.39 (95% CI 0.13-1.17); proportion of participants with laboratory confirmed secondary influenza episode | Adjusted OR: 0.61 (95% CI 0.2-1.87); proportion of participants with ILI | Proportion of contacts in control and intervention group developing ILI [28/53 (53%) vs 11/36 (31%)], p= 0.04 |
|  | Sources of information used/obtained to help inform risk of bias judgement | Journal article(s) with results of the trial | Journal article(s) with results of the trial | Journal article(s) with results of the trial, trial protocol | Journal article(s) with results of the trial, trial protocol |  |  | Journal article(s) with results of the trial |
| Bias Domain | Signalling Question | Responses | | | | | | |
| Bias arising from the randomization process | 1a.1 Was the allocation sequence random? | Y | Y | Y | Y | Y | Y | NI |
|  | Description/Support for judgement | Quote: "The residence hall units were randomized by blindly selecting a uniform ticket with the name of each hall out of a container (A.S.M. and A.A.) for randomization assignment to each study arm" | Quote: "Enrolled families were randomized to one of the three study arms in a 1:1:1 ratio. Randomization was achieved using a block randomization method using a list of blocks each with 12 household IDs"  Comment: Block sizes are small and of equal size (n=4) across the intervention arms, and a study staff was assigned allocate the subjects, suggesting that allocation sequence was not adequately concealed | Quote: "Text S1: Residence houses were contained within each of the five residence halls in the study (n = 5), thus ensuring that each residence hall had residence houses assigned to each intervention and control arm. …(until)..Residence houses were contained within each of the five residence halls in the study (n = 5), thus ensuring that each residence hall had residence houses assigned to each intervention and control arm." | "Text S1: Residence houses were contained within each of the five residence halls in the study (n = 5), thus ensuring that each residence hall had residence houses assigned to each intervention and control arm. …(until)..Residence houses were contained within each of the five residence halls in the study (n = 5), thus ensuring that each residence hall had residence houses assigned to each intervention and control arm." | Quote:"We used a cluster randomisation with the households serving as clusters. We prepared lists of random numbers with Microsoft Excel 2003 (Mircosoft™ Cooperation, Seattle, USA) which were divided between the three intervention groups. Each participating physician received a list of random numbers with the interventions represented in a 1:1:1 ratio. Eligible index patients were randomly assigned a number, which was then communicated to the study center." | Quote:"We used a cluster randomisation with the households serving as clusters. We prepared lists of random numbers with Microsoft Excel 2003 (Mircosoft™ Cooperation, Seattle, USA) which were divided between the three intervention groups. Each participating physician received a list of random numbers with the interventions represented in a 1:1:1 ratio. Eligible index patients were randomly assigned a number, which was then communicated to the study center." | Quote: "The tent was the randomisation unit, and tents were randomised to either intervention group (supervised mask tent) or control group (no supervised mask tent) by an independent study coordinator who was not an investigator." Judgement: no information on how the allocation sequence used by the study coordinator allocating groups was generated |
|  | 1a.2 Is it likely that the allocation sequence was subverted? | PN | PN | PN | PN | N | N | PN |
|  | Description/Support for judgement | Quote: "The largest of the 7 residence halls housed 1240 residents. The 6 smaller residence halls ranged from 110 to 830 residents. The 6 smaller halls were combined into 2 similar sized units, to create a comparable size to the largest residence hall; all 3 similar sized units were then randomized to the intervention or control arms. The residence hall units were randomized by blindly selecting a uniform ticket with the name of each hall out of a container (A.S.M. and A.A.) for randomization assignment to each study arm" Comment: Residence halls were combined on the basis of overall cluster size generated, and the clusters were randomised by drawing lots of equal size. hence subversion of allocation sequence is not likely | Quote:"A study coordinator assigned each household to one study arm after consent was obtained. Recruiting clinicians were blinded to the allocation of the specific intervention. ", Table 1 Comment: Individuals are likely to not be aware of potential allocations prior to their allocation as parties assigning allocations are not the same as the recruiting parties. Table 1 also shows that there were no baseline imbalances between groups | Quote:"Text S1: Residence houses were contained within each of the five residence halls in the study (n = 5), thus ensuring that each residence hall had residence houses assigned to each intervention and control arm. …(until)..Residence houses were contained within each of the five residence halls in the study (n = 5), thus ensuring that each residence hall had residence houses assigned to each intervention and control arm." | Quote:"Text S1: Residence houses were contained within each of the five residence halls in the study (n = 5), thus ensuring that each residence hall had residence houses assigned to each intervention and control arm. …(until)..Residence houses were contained within each of the five residence halls in the study (n = 5), thus ensuring that each residence hall had residence houses assigned to each intervention and control arm." | Quote: "The resulting intervention was only communicated to the households with the physicians (as well as laboratory personnel) blinded from the randomisation results. Intervention material was given to the study sites in closed boxes marked only with the randomisation number. Recruiting physicians were not aware of the allocation of the numbers to the interventions and the boxes for the three intervention arms looked identical. After randomisation, participants were given their box by the physician's assistants." Comment: Subversion was not likely to have occurred since recruiting physicians were not aware of allocation sequence | Quote: "The resulting intervention was only communicated to the households with the physicians (as well as laboratory personnel) blinded from the randomisation results. Intervention material was given to the study sites in closed boxes marked only with the randomisation number. Recruiting physicians were not aware of the allocation of the numbers to the interventions and the boxes for the three intervention arms looked identical. After randomisation, participants were given their box by the physician's assistants." Comment: Subversion was not likely to have occurred since recruiting physicians were not aware of allocation sequence | Quote: The tent was the randomisation unit, and tents were randomised to either intervention group (supervised mask tent) or control group (no supervised mask tent) by an independent study coordinator who was not an investigator. Comment: Randomisation and recruitment were done by different people. |
|  | 1a.3 Were there baseline imbalances that suggest a problem with the randomization process? | N | N | N | N | N | N | N |
|  | Description/Support for judgement | Table 1 | Table 1b | Quote: Table 1 Comment: The numbers are equally distributed between the arms (349 vs 392 vs 370) and there is no significant difference at baseline (table S1) | Quote: Table 1 Comment: The numbers are equally distributed between the arms (349 vs 392 vs 370) and there is no significant difference at baseline (table S1) | Quote: Table 1, "Table 1 shows the baseline characteristics of index patients and household contacts of all analyzed households stratified by season of study participation and intervention group. One difference between the two study seasons was that in 2009/10 all viruses belonged to A (H1N1) pdm09, while in 2010/11 both A (H1N1) pdm09 as well as B viruses circulated (p = 0.14 for comparison within season). Another difference is that - compared to 2009/10 - the number of index patients receiving timely antiviral therapy was significantly higher in 2010/11 (p < 0.001). Furthermore, in 2010/11 a significantly larger number of both index patients (p = 0.045) and household contacts (p = 0.003) was vaccinated compared to 2009/10. Finally, in 2009/10 randomisation occurred significantly earlier after symptom onset compared to 2010/11 (p = 0.004) and a higher proportion of households was visited by study personnel within 36 h (p = 0.04). All other variables did not differ significantly between the two study seasons (Table 1)" Comment: Observed baseline imbalances were likely to have arose due to chance rather than a problem with randomisation | Quote: Table 1, "Table 1 shows the baseline characteristics of index patients and household contacts of all analyzed households stratified by season of study participation and intervention group. One difference between the two study seasons was that in 2009/10 all viruses belonged to A (H1N1) pdm09, while in 2010/11 both A (H1N1) pdm09 as well as B viruses circulated (p = 0.14 for comparison within season). Another difference is that - compared to 2009/10 - the number of index patients receiving timely antiviral therapy was significantly higher in 2010/11 (p < 0.001). Furthermore, in 2010/11 a significantly larger number of both index patients (p = 0.045) and household contacts (p = 0.003) was vaccinated compared to 2009/10. Finally, in 2009/10 randomisation occurred significantly earlier after symptom onset compared to 2010/11 (p = 0.004) and a higher proportion of households was visited by study personnel within 36 h (p = 0.04). All other variables did not differ significantly between the two study seasons (Table 1)" Comment: Observed baseline imbalances were likely to have arose due to chance rather than a problem with randomisation | Quote: Table 1 Comment: groups did not have significant differences in most reported demographics, unless there were demographics collected that were not reported in the table, which we would not know as the content of the questionnaire is not detailed. |
|  | **Risk of bias judgement** | **High** | **High** | **High** | **High** | **High** | **High** | **High** |
|  | General Note | Allocation sequence was randomly generated and unlikely subverted as it was done using the drawing of lots, baseline imbalances observed does not suggest a problem with the randomisation process |  | Allocation sequence was randomly generated and unlikely subverted based on protocol reported, baseline imbalances observed does not suggest a problem with the randomisation process | Allocation sequence was randomly generated and unlikely subverted based on protocol reported, baseline imbalances observed does not suggest a problem with the randomisation process | Allocation sequence was randomly generated and unlikely subverted based on protocol reported, baseline imbalances observed does not suggest a problem with the randomisation process | Allocation sequence was randomly generated and unlikely subverted based on protocol reported, baseline imbalances observed does not suggest a problem with the randomisation process |  |
|  | Optional: What is the predicted direction of bias arising from the randomization process? | Nil | Nil | Nil | Nil | Nil | Nil | Nil |
|  | Description/Support for judgement | Nil | Nil | Nil | Nil | Nil | Nil | Nil |
| Bias arising from the timing of identification and recruitment of individual participants in relation to timing of randomization | 1b.1 Were all the individual participants identified before randomization of clusters (and if the trial specifically recruited patients were they all recruited before randomization of clusters)? | PN | NI | N | N | N | N | N |
|  | Description/Support for judgement | Quote: "The residence hall units were randomized by blindly selecting a uniform ticket with the name of each hall out of a container (A.S.M. and A.A.) for randomization assignment to each study arm. The largest single residence hall was randomized to the mask plus alcohol-based hand sanitizer (62% ethyl alcohol in a gel base) group (hereafter, the “face mask and hand hygiene” group), a cluster composed of 4 residence halls was randomized to the face mask–only group, and the remaining 2 residence halls served as the control group. We estimated a sample size of 750 participants per intervention group...Students living in these residence halls were eligible for participation if they were at least 18 years of age..." Comment: Likely that subjects were enrolled from the respective halls onyl after randomisation was done | Quote:"Enrolled families were randomized to one of the three study arms in a 1:1:1 ratio. Randomization was achieved using a block randomization method using a list of blocks each with 12 household IDs, four of which were assigned to each of the three study arms. A study coordinator assigned each household to one study arm after consent was obtained. Recruiting clinicians were blinded to the allocation of the specific intervention" Comment: Participants were not directly recruited, but it is unclear whether they were recruited prior to randomisation based on reported methods, although the equal number of clusters in each intervention arm suggests they are likely to have been randomised after recruitment | Quote: "Thirty-seven residence houses located in five residence halls were randomly assigned to either an intervention or a control group. All residence houses in each of the residence halls were randomized prior to the intervention implementation." Comment: Stated that randomisation was carried out before identification | Quote: "Thirty-seven residence houses located in five residence halls were randomly assigned to either an intervention or a control group. All residence houses in each of the residence halls were randomized prior to the intervention implementation." Comment: Stated that randomisation was carried out before identification | Quote: "We prepared lists of random numbers with Microsoft Excel 2003 (Mircosoft™ Cooperation, Seattle, USA) which were divided between the three intervention groups. Each participating physician received a list of random numbers with the interventions represented in a 1:1:1 ratio. Eligible index patients were randomly assigned a number, which was then communicated to the study center. The resulting intervention was only communicated to the households with the physicians (as well as laboratory personnel) blinded from the randomisation results." | Quote: "We prepared lists of random numbers with Microsoft Excel 2003 (Mircosoft™ Cooperation, Seattle, USA) which were divided between the three intervention groups. Each participating physician received a list of random numbers with the interventions represented in a 1:1:1 ratio. Eligible index patients were randomly assigned a number, which was then communicated to the study center. The resulting intervention was only communicated to the households with the physicians (as well as laboratory personnel) blinded from the randomisation results." | Quote:"Study brochures were distributed to Australian pilgrims in Mosques, Islamic Centres and pre-travel seminars in Australia and during their stay in hotels in Mecca, Saudi Arabia before they travelled to their tents in the Mina valley, located at the outskirts of Mecca", "The tent was the randomisation unit, and tents were randomised to either intervention group (supervised mask tent) or control group (no supervised mask tent) by an independent study coordinator who was not an investigator.", "Australian pilgrims who had ILI symptoms for 3 days or less were invited to participate as a ‘case’. People who slept in an adjacent bed were invited to participate as a ‘contact’." Comment: Subjects were likely idntified and recruited after clusters were randomised |
|  | 1b.2 If N/PN/NI to 1b.1: Is it likely that selection of individual participants was affected by knowledge of the intervention? | NI | N | PY | PY | N | N | N |
|  | Description/Support for judgement | No information provided on whether the recruitment was carried out by research team (who will have knowledge of the allocation) or other parties who are unaware of the allocation. | Quote:"A study coordinator assigned each household to one study arm after consent was obtained. Recruiting clinicians were blinded to the allocation of the specific intervention" Comment: Separate parties were involved in randomisation and recruitment. | Quote:"Recruitment by study staff…", "Recruiting staff are taught to avoid coercion or undue influence when recruiting and enrolling potential participants.(in Protocol S1)" Comment: The study staff who might be aware of the allocation subconsciously differentially include potential individual participants in different trial arms | Quote:"Recruitment by study staff…", "Recruiting staff are taught to avoid coercion or undue influence when recruiting and enrolling potential participants.(in Protocol S1)" Comment: The study staff who might be aware of the allocation subconsciously differentially include potential individual participants in different trial arms | Quote: "Recruiting physicians were not aware of the allocation of the numbers to the interventions and the boxes for the three intervention arms looked identical" | Quote: "Recruiting physicians were not aware of the allocation of the numbers to the interventions and the boxes for the three intervention arms looked identical" | Quote: "The tent was the randomisation unit, and tents were randomised to either intervention group (supervised mask tent) or control group (no supervised mask tent) by an independent study coordinator who was not an investigator."  Comment: unlikely as recruitment and randomization by different people |
|  | 1b.3 Were there baseline imbalances that suggest differential identification or recruitment of individual participants between arms? | PY | N | N | N | N | N | N |
|  | Description/Support for judgement | Quote: Table 1 Comment: Imbalance of subject numbers in control group, compared to the intervention arms which is posibly due to recruitment bias as there is no information of the parties involved in recruitment and the methods used for randomisation was unlikely to result in bias | Quote: Table 1 Comment: numbers of participants and baseline characteristics of participants are balanced across arms | Quote: Table 1 Comment: The numbers are equally distributed between the arms (349 vs 392 vs 370) and there is no significant difference at baseline (table S1) | Quote: Table 1 Comment: The numbers are equally distributed between the arms (349 vs 392 vs 370) and there is no significant difference at baseline (table S1) | Quote: Table 1, "Table 1 shows the baseline characteristics of index patients and household contacts of all analyzed households stratified by season of study participation and intervention group. One difference between the two study seasons was that in 2009/10 all viruses belonged to A (H1N1) pdm09, while in 2010/11 both A (H1N1) pdm09 as well as B viruses circulated (p = 0.14 for comparison within season). Another difference is that - compared to 2009/10 - the number of index patients receiving timely antiviral therapy was significantly higher in 2010/11 (p < 0.001). Furthermore, in 2010/11 a significantly larger number of both index patients (p = 0.045) and household contacts (p = 0.003) was vaccinated compared to 2009/10. Finally, in 2009/10 randomisation occurred significantly earlier after symptom onset compared to 2010/11 (p = 0.004) and a higher proportion of households was visited by study personnel within 36 h (p = 0.04). All other variables did not differ significantly between the two study seasons (Table 1)" Comment: Observed baseline imbalances were likely to have arose due to chance rather than a problem with randomisation | Quote: Table 1, "Table 1 shows the baseline characteristics of index patients and household contacts of all analyzed households stratified by season of study participation and intervention group. One difference between the two study seasons was that in 2009/10 all viruses belonged to A (H1N1) pdm09, while in 2010/11 both A (H1N1) pdm09 as well as B viruses circulated (p = 0.14 for comparison within season). Another difference is that - compared to 2009/10 - the number of index patients receiving timely antiviral therapy was significantly higher in 2010/11 (p < 0.001). Furthermore, in 2010/11 a significantly larger number of both index patients (p = 0.045) and household contacts (p = 0.003) was vaccinated compared to 2009/10. Finally, in 2009/10 randomisation occurred significantly earlier after symptom onset compared to 2010/11 (p = 0.004) and a higher proportion of households was visited by study personnel within 36 h (p = 0.04). All other variables did not differ significantly between the two study seasons (Table 1)" Comment: Observed baseline imbalances were likely to have arose due to chance rather than a problem with randomisation | Quote: Table 1 Comment: groups did not have significant differences in most reported demographics, unless there were demographics collected that were not reported in the table, which we would not know as the content of the questionnaire is not detailed. |
|  | **Risk of bias judgement** | **High** | **Low** | **High** | **High** | **Low** | **Low** | **Low** |
|  | General Note | High risk of bias arising from timing of identification or recruitment as participants were likely recruited after clusters were randomised, there was no information on whether the recruiting parties were aware of intervention allocation but baseline imbalances across groups suggest the likelihood of recruitment bias as the randomisation methods used were unlikely to hav resulted in bias | Bias arising from timing of identification/ recruitment as selection of individual participants was not affected by knowledge of intervention allocation and there was no evidence suffesting differential identificaiton or recruitment of participants between arms | High risk of bias arising from timing of identification or recruitment as participants were recruited after clusters were randomised and the recruiting parties were aware of intervention allocation, although there were no baseline imbalances across groups suggesting the presence of recruitment bias | High risk of bias arising from timing of identification or recruitment as participants were recruited after clusters were randomised and the recruiting parties were aware of intervention allocation, although there were no baseline imbalances across groups suggesting the presence of recruitment bias | Bias arising from timing of identification/ recruitment as selection of individual participants was not affected by knowledge of intervention allocation and there was no evidence suffesting differential identificaiton or recruitment of participants between arms | Bias arising from timing of identification/ recruitment as selection of individual participants was not affected by knowledge of intervention allocation and there was no evidence suffesting differential identificaiton or recruitment of participants between arms | Bias arising from timing of identification/ recruitment as selection of individual participants was not affected by knowledge of intervention allocation and there was no evidence suffesting differential identification or recruitment of participants between arms |
|  | Optional: What is the predicted direction of bias arising from the timing of identification and recruitment of individual participants? | Nil | Nil | Nil | Nil | Nil | Nil | Nil |
|  | Description/Support for judgement | Nil | Nil | Nil | Nil | Nil | Nil | Nil |
| Bias due to deviations from intended interventions | 2.1a Were participants aware that they were in a trial? | PY | PY | PY | PY | PY | PY | PY |
|  | Description/Support for judgement | Quote: "Written informed consent was obtained from all participants." Comment: Possible since informed consent was obtained, but we do not have access to recruitment materials used to determine if the subjects were briefed of other possible interventions, or just their intervention since the clusters were likely randomised prior to recruitment. Nonetheless the US regulations for human subjects states that informed consent includes "a description of the procedures to be followed, and identification of any procedures which are experimental;" | Quote: "All subjects aged 18 years and older provided written consent to participate, and proxy written consent from parents or legal guardians was obtained for children. Households were compensated for their time to participate in the study with approximately US $60 in Thai baht. " Comment: Highly likely since consent was obtained and compensation was given for participation | Quote: "Consents were taken from the students.", "Recruitment efforts are designed to comply with IRB approved methods. Research subjects are never induced to participate by means other than voluntary consent and with knowledge of the research study and its requirements." Comment: Possible since informed consent was obtained, but we do not have access to recruitment materials used (website linking to recruitment materials in Procotol S1 has expired) to determine if the subjects were briefed of other possible interventions, or just their intervention since the clusters were likely randomised prior to recruitment. Nonetheless the US regulations for human subjects states that informed consent includes "a description of the procedures to be followed, and identification of any procedures which are experimental" | Quote: "Consents were taken from the students.", "Recruitment efforts are designed to comply with IRB approved methods. Research subjects are never induced to participate by means other than voluntary consent and with knowledge of the research study and its requirements." Comment: Possible since informed consent was obtained, but we do not have access to recruitment materials used (website linking to recruitment materials in Procotol S1 has expired) to determine if the subjects were briefed of other possible interventions, or just their intervention since the clusters were likely randomised prior to recruitment. Nonetheless the US regulations for human subjects states that informed consent includes "a description of the procedures to be followed, and identification of any procedures which are experimental" | Quote: "We obtained written informed consent from all study participants. If these were less than 18 years of age we asked their parents or legal guardians to provide proxy written consent, with additional written consent from those participants aged 14 to 18 years of age. Children were defined as persons aged less than 14 years, adults were at least 14 years old." Comment: Possible since informed consent was obtained, but we do not have access to recruitment materials used to determine if the subjects were briefed of other possible interventions during consent taking, or just their intervention since the clusters were likely randomised prior to recruitment. Nonetheless the US regulations for human subjects states that informed consent includes "a description of the procedures to be followed, and identification of any procedures which are experimental" It is likely that they are aware that they are part of a trial | Quote: "We obtained written informed consent from all study participants. If these were less than 18 years of age we asked their parents or legal guardians to provide proxy written consent, with additional written consent from those participants aged 14 to 18 years of age. Children were defined as persons aged less than 14 years, adults were at least 14 years old." Comment: Possible since informed consent was obtained, but we do not have access to recruitment materials used to determine if the subjects were briefed of other possible interventions during consent taking, or just their intervention since the clusters were likely randomised prior to recruitment. Nonetheless the US regulations for human subjects states that informed consent includes "a description of the procedures to be followed, and identification of any procedures which are experimental" It is likely that they are aware that they are part of a trial | Quote:"Study brochures were distributed to Australian pilgrims in Mosques, Islamic Centres and pre-travel seminars in Australia and during their stay in hotels in Mecca, Saudi Arabia before they travelled to their tents in the Mina valley, located at the outskirts of Mecca", "Australian pilgrims who had ILI symptoms for 3 days or less were invited to participate as a ‘case’. People who slept in an adjacent bed were invited to participate as a ‘contact’." Pilgrims in control tents seems not know that they are in the control group, as they were merely "not provided with facemasks" but not asked to not wear facemasks." Comment: Possible since informed consent was obtained, but we do not have access to recruitment materials used to determine if the subjects were briefed of other possible interventions during consent taking, or just their intervention since the clusters were likely randomised prior to recruitment. Nonetheless the Australian regulations for human subjects states that informed consent includes "An information and consent document or other consent strategy should be appropriate to the needs of the participants and proportional to the project’s risks and ethical sensitivity (Section 3.1.3)" It is likely that they are aware that they are part of a trial |
|  | 2.1b If Y/PY/NI to 2.1a: Were participants aware of their assigned intervention during the trial? | Y | Y | Y | Y | Y | Y | PY |
|  | Description/Support for judgement | Quote: "All participants received basic hand hygiene education (proper hand hygiene practices and cough etiquette) through an email video link and the study Web site. In addition, face mask and hand hygiene group participants received written materials detailing appropriate hand sanitizer and mask use; ..(all the way till)... Mask and hand hygiene group participants also received alcohol-based hand sanitizer (portable 2 oz squeeze bottle; 8 oz pump) for use throughout the study", " Because of the inability to blind participants to study interventions, compliance with these interventions must be considered carefully" Comment: Nature of intervention made it impossible to blind participants to their intervention | Quote": "The control group received nutritional, physical activity, and smoking cessation education. Intervention group 1 households received hand-washing education and a hand-washing ... Intervention group 2 households received hand-washing education and the hand-washing kit, and a box of 50 standard paper surgical face masks and 20 pediatric face masks" Comment: nature of intervention made it impossible to blind the participants | Quote: "The intervention groups included mask and hand hygiene or mask alone. Participants in the face mask and hand hygiene and the face mask only groups received weekly packets of mask supplies in their student mailboxes. ...Participants were asked to wear their masks for at least six hours per day while in their residence hall. Students were encouraged but not obligated to wear their face masks outside of their residence hall. In addition to masks, all participants in the face mask and hand hygiene intervention received hand sanitizer. The control group did not receive an intervention." Comment: Nature of intervention made it impossible to blind participants to their intervention | Quote: "The intervention groups included mask and hand hygiene or mask alone. Participants in the face mask and hand hygiene and the face mask only groups received weekly packets of mask supplies in their student mailboxes. ...Participants were asked to wear their masks for at least six hours per day while in their residence hall. Students were encouraged but not obligated to wear their face masks outside of their residence hall. In addition to masks, all participants in the face mask and hand hygiene intervention received hand sanitizer. The control group did not receive an intervention." Comment: Nature of intervention made it impossible to blind participants to their intervention | Quote: "Intervention material was given to the study sites in closed boxes marked only with the randomisation number.", "The following three intervention groups were used: (i) Mask/Hygiene (MH) arm: households were provided with alcohol based hand-rub (Sterilium™, Bode Chemie, Germany) and surgical facemasks in two different sizes, one for children aged younger than 14 years (Child’s Face Mask, Kimberly-Clark, USA) and one for adults (Aérokyn Masques, LCH Medical Products, France). If masks intended for participants younger than 14 years did not fit properly (as assessed by study personnel during the first household visit), we asked them to wear adult masks instead. Household also received information on the proper use of the interventions; (ii) Mask (M) arm: we provided the household with surgical facemasks and information on their correct use; (iii) Control (C) arm: no masks or hand rub was provided." Comment: Nature of intervention made it impossible to blind participants to their intervention | Quote: "Intervention material was given to the study sites in closed boxes marked only with the randomisation number.", "The following three intervention groups were used: (i) Mask/Hygiene (MH) arm: households were provided with alcohol based hand-rub (Sterilium™, Bode Chemie, Germany) and surgical facemasks in two different sizes, one for children aged younger than 14 years (Child’s Face Mask, Kimberly-Clark, USA) and one for adults (Aérokyn Masques, LCH Medical Products, France). If masks intended for participants younger than 14 years did not fit properly (as assessed by study personnel during the first household visit), we asked them to wear adult masks instead. Household also received information on the proper use of the interventions; (ii) Mask (M) arm: we provided the household with surgical facemasks and information on their correct use; (iii) Control (C) arm: no masks or hand rub was provided." Comment: Nature of intervention made it impossible to blind participants to their intervention | Quote:"Study brochures were distributed to Australian pilgrims in Mosques, Islamic Centres and pre-travel seminars in Australia and during their stay in hotels in Mecca, Saudi Arabia before they travelled to their tents in the Mina valley, located at the outskirts of Mecca", "Australian pilgrims who had ILI symptoms for 3 days or less were invited to participate as a ‘case’. People who slept in an adjacent bed were invited to participate as a ‘contact’." Pilgrims in control tents seems not know that they are in the control group, as they were merely "not provided with facemasks" but not asked to not wear facemasks." Comment: Possible since informed consent was obtained, but we do not have access to recruitment materials used to determine if the subjects were briefed of other possible interventions during consent taking, or just their intervention since the clusters were likely randomised prior to recruitment. Nonetheless the Australian regulations for human subjects states that informed consent includes "An information and consent document or other consent strategy should be appropriate to the needs of the participants and proportional to the project’s risks and ethical sensitivity (Section 3.1.3)" It is likely that they are aware that they are part of a trial |
|  | 2.2. Were carers and trial personnel aware of participants' assigned intervention during the trial? | PY | Y | PY | PY | N | N | Y |
|  | Description/Support for judgement | Quote: "The residence hall units were randomized by blindly selecting a uniform ticket with the name of each hall out of a container (A.S.M. and A.A.) for randomization assignment to each study arm. The largest single residence hall was randomized to the mask plus alcohol-based hand sanitizer (62% ethyl alcohol in a gel base) group (hereafter, the “face mask and hand hygiene” group), a cluster composed of 4 residence halls was randomized to the face mask–only group, and the remaining 2 residence halls served as the control group. We estimated a sample size of 750 participants per intervention group...Students living in these residence halls were eligible for participation if they were at least 18 years of age...", "All participants received basic hand hygiene education (proper hand hygiene practices and cough etiquette) through an email video link and the study Web site. In addition, face mask and hand hygiene group participants received written materials detailing appropriate hand sanitizer and mask use; ..(all the way till)... Mask and hand hygiene group participants also received alcohol-based hand sanitizer (portable 2 oz squeeze bottle; 8 oz pump) for use throughout the study" Comment: Randomisation was completed before recruitment and there was insufficient information to decide whether trial personnel were not aware of the assigned intervention (though it is highly likely they were); nature of intervention made it impossible to blind participants to their intervention | Quote": "The control group received nutritional, physical activity, and smoking cessation education. Intervention group 1 households received hand-washing education and a hand-washing ... Intervention group 2 households received hand-washing education and the hand-washing kit, and a box of 50 standard paper surgical face masks and 20 pediatric face masks" Comment: nature of intervention made it impossible to blind the participants | Quote: "Intervention materials and a required educational video on proper hand hygiene and use of standard medical procedure face masks were provided to study participants on January 24th.", "Text S1: Study-staffed tables located in each residence hall offered a surplus of face masks and hand sanitizer (with exchange of used, empty bottles) to ensure a supply chain of intervention materials. As with mask packets, participants’ signatures were logged to confirm receipt of surplus supplies." Comment: It is highly possible that the trial personnel have to know the assigned intervention to distribute the intervention | Quote: "Intervention materials and a required educational video on proper hand hygiene and use of standard medical procedure face masks were provided to study participants on January 24th.", "Text S1: Study-staffed tables located in each residence hall offered a surplus of face masks and hand sanitizer (with exchange of used, empty bottles) to ensure a supply chain of intervention materials. As with mask packets, participants’ signatures were logged to confirm receipt of surplus supplies." Comment: It is highly possible that the trial personnel have to know the assigned intervention to distribute the intervention | Quote: "The resulting intervention was only communicated to the households with the physicians (as well as laboratory personnel) blinded from the randomisation results. Recruiting physicians were not aware of the allocation of the numbers to the interventions and the boxes for the three intervention arms looked identical. After randomisation, participants were given their box by the physician's assistants." | Quote: "The resulting intervention was only communicated to the households with the physicians (as well as laboratory personnel) blinded from the randomisation results. Recruiting physicians were not aware of the allocation of the numbers to the interventions and the boxes for the three intervention arms looked identical. After randomisation, participants were given their box by the physician's assistants." | Quote: "For intervention tent groups, masks were provided to both index cases and their contacts. Advice on mask use was given throughout their stay in Mina: before prayers, in seminars and after meals. Written instructions were provided on how to use facemasks, when to change them and when (i.e. if damp or damaged) and where (a special polythene bag) to discard used masks. Pilgrims allocated to control tents were not provided with facemasks but general information on hygiene was given to them." |
|  | 2.3. If Y/PY/NI to 2.1 or 2.2: Were there deviations from the intended intervention beyond what would be expected in usual practice? | NI | PY | PN | PN | PY | PY | NI |
|  | Description/Support for judgement | Quote: "Additional results on survey-reported and observed compliance are presented in Section A2 of the Appendix." Comment: Appendix not available online. Review authors have reached out to corresponding author for information but have not received any response. Thus, there are is insufficient information to determine if any deviations occurred, absed on what was presented in the publication | Quote: "In an analysis of hand-washing behavior of 207 control group subjects enrolled before June 1, 2009 and 162 enrolled after that date (the approximate onset of the first wave of the 2009 pandemic), mean reported handwashing episodes per day increased from 3.7 to 4.1 (P = 0.09). Mean reported daily face mask use also increased during June to August 2009. When asked during the Day 7 home visit, 65 of 370 (17.6%) control family members reported using used facemasks during the study week and 44 (67.7%) of these were members of families enrolled after June 1, 2009....Among index cases in the control arm, 3 of 83 (4%) enrolled before the pandemic reported using a mask during the study week, compared to 29 of 56 (52%) of index patients enrolled after June 1, 2009 (P <0.001)." Comment: Deviations are likely to have occurred due to external factors and trial context (due to the pandemic and perhaps even as a result of the basic hand hygiene education) | Quote: "Compliance" Section, Section III in Text S1 Comment: Text indicated that the participants complied to the interventions they were assigned to | Quote: "Compliance" Section, Section III in Text S1 Comment: Text indicated that the participants complied to the interventions they were assigned to | Quote: "We observed “contamination” between intervention groups in two control households only in the season 2009/10, one reported wearing masks, the other reported wearing masks and using alcohol based hand sanitizer. Examination of further potentially relevant behavioural variables, such as daily time spent at home during the study period, time spent at close range of the index patient, sleeping in the same room or taking meals with the index patient, did not result in significant differences between the study groups or between influenza seasons (data not shown)" Comment: Deviations may have occurred due to trial context | Quote: "We observed “contamination” between intervention groups in two control households only in the season 2009/10, one reported wearing masks, the other reported wearing masks and using alcohol based hand sanitizer. Examination of further potentially relevant behavioural variables, such as daily time spent at home during the study period, time spent at close range of the index patient, sleeping in the same room or taking meals with the index patient, did not result in significant differences between the study groups or between influenza seasons (data not shown)" Comment: Deviations may have occurred due to trial context | Quote: "Compliance with facemask use by pilgrims in the ‘mask’ group was 56 of 75 (76%), while it was 11 of 89 (12%) in the ‘control’ group (p<0.001). The proportion of facemask user in the ‘mask’ tents was 76% for both males (19/25) and females (38/50). The most often reported reason for not wearing facemasks was discomfort (15%)." Comment: More information is needed on whether deviations from the intended interventions arose due to trial context |
|  | 2.4. If Y/PY to 2.3: Were these deviations from intended intervention unbalanced between groups *and* likely to have affected the outcome? | N/A | N | N/A | N/A | PY | PY | N/A |
|  | Description/Support for judgement | Nil | Quote: "Subjects in the control arm reported an average of 3.9 hand-washing episodes ⁄ day (on Day 7) while subjects in the hand washing arm reported an average of 4.7 handwashing episodes ⁄ day (95% CI 4.3–5.0; P = 0.002 compared to controls), and subjects in the hand washing plus face mask arm reported 4.9 episodes ⁄ day (95% CI 4.5–5.3; P < 0.00011 compared to controls" Comment: Unlikely since there was still a significant difference in NPI practised between intervention and control groups | The type III fixed effects model for assessing differences over time using a week * group interaction term, was not statistically significant. (Fig 2) | There is a significant finding in the face mask+hygiene VS control in wk 3-6 and not in week 1 and 2 | Quote: "We observed “contamination” between intervention groups in two control households only in the season 2009/10, one reported wearing masks, the other reported wearing masks and using alcohol based hand sanitizer. Examination of further potentially relevant behavioural variables, such as daily time spent at home during the study period, time spent at close range of the index patient, sleeping in the same room or taking meals with the index patient, did not result in significant differences between the study groups or between influenza seasons (data not shown)" Comment: Deviations occurred only in control group, and would have biased the summary measure obtained towards the null should the NPIs be truly effective in preventing secondary influenza episodes | Quote: "We observed “contamination” between intervention groups in two control households only in the season 2009/10, one reported wearing masks, the other reported wearing masks and using alcohol based hand sanitizer. Examination of further potentially relevant behavioural variables, such as daily time spent at home during the study period, time spent at close range of the index patient, sleeping in the same room or taking meals with the index patient, did not result in significant differences between the study groups or between influenza seasons (data not shown)" Comment: Deviations occurred only in control group, and would have biased the summary measure obtained towards the null should the NPIs be truly effective in preventing secondary influenza episodes | Nil |
|  | 2.5a Were any clusters analysed in a group different from the one to which they were assigned? | N | N | N | N | N | N | N |
|  | Description/Support for judgement | Quote: "The total number of participants analyzed was 1297 with 367 in the face mask and hand hygiene group (9 deemed ineligible and 26 lost to follow-up), 378 in the face mask–only group (11 deemed ineligible and 52 lost to follow-up), and 552 in the control group (19 deemed ineligible and 21 lost to follow-up) (Figure A1 in the Appendix). In total, 1297 (97%) of 1331 participants completed a baseline and at least 1 weekly survey" Comment: All subjects were analysed according to the group they were assigned to | Quote: Intention to treat analysis section | Intention to treat analysis | Intention to treat analysis | Quote: "Apart from these exclusions of non-adherent study participants, we conducted the same analyses as for the intention-to-treat analysis. All OR of the M and MH group were below 1, mostly between 0.2 and 0.3 relating to a protective effect of 70%-80% for the interventions (Table ​5). Significant results were reached in the M group when analysing the complete data set and in the M as well as the MH group when considering only A(H1N1)pdm09 households." | Quote: "Apart from these exclusions of non-adherent study participants, we conducted the same analyses as for the intention-to-treat analysis. All OR of the M and MH group were below 1, mostly between 0.2 and 0.3 relating to a protective effect of 70%-80% for the interventions (Table ​5). Significant results were reached in the M group when analysing the complete data set and in the M as well as the MH group when considering only A(H1N1)pdm09 households." | Quote: "A total of 164 pilgrims were recruited; 75 in the ‘mask’ group (39 ‘cases’ and 36 ‘contacts’), and 89 in the ‘control’ group (36 ‘cases’ and 53 ‘contacts’). ", Table 1, Figure 1 Cpmment:Clusters were analysed in groups they were assigned to |
|  | 2.5b Were any participants analysed in a group different from the one to which their original cluster was randomized? | N | N | N | N | N | N | N |
|  | Description/Support for judgement | Quote: "The total number of participants analyzed was 1297 with 367 in the face mask and hand hygiene group (9 deemed ineligible and 26 lost to follow-up), 378 in the face mask–only group (11 deemed ineligible and 52 lost to follow-up), and 552 in the control group (19 deemed ineligible and 21 lost to follow-up) (Figure A1 in the Appendix). In total, 1297 (97%) of 1331 participants completed a baseline and at least 1 weekly survey" Comment: All subjects were analysed according to the group they were randomised to | Quote: Intention to treat analysis section | Intention to treat analysis | Intention to treat analysis | Intention to treat analysis | Intention to treat analysis | Table 1, Intention to treat analysis |
|  | 2.6 If Y/PY/NI to 2.5: Was there potential for a substantial impact (on the estimated effect of intervention) of analysing participants in the wrong group? | N/A | N/A | N/A | N/A | N/A | N/A | N/A |
|  | Description/Support for judgement | Nil | Nil | Nil | Nil | Nil | Nil | Nil |
|  | **Risk of bias judgement** | **Some concerns** | **Low** | **Low** | **Low** | **High** | **High** | **Some Concerns** |
|  | General Note | Participants and people delivering the interventions were likely aware of intervention groups during the trial and clusters were analysed in their original group of assignment and randomisation but there was not enough information to determine if there was any deviation from trial protocol beyonf what was expected from usual practice | Participants and people delivering the interventions were aware of intervention groups during the trial and deviations from intended intervention arose because of trial context but did not affect the outcome and clustrs were analysed in their original group of assignment and randomisation | Participants and people delivering the interventions were aware of intervention groups during the trial and there were no reported deviations from intended intervention that arose because of trial context and clusters were analysed in their original group of assignment and randomisation | Participants and people delivering the interventions were aware of intervention groups during the trial and there were no reported deviations from intended intervention that arose because of trial context and clusters were analysed in their original group of assignment and randomisation | Participants and people delivering the interventions were aware of intervention groups during the trial and there were reported deviations from intended intervention that arose because of trial context (and may are likely to have affected the outcome), but clusters were analysed in their original group of assignment and randomisation | Participants and people delivering the interventions were aware of intervention groups during the trial and there were reported deviations from intended intervention that arose because of trial context (and may are likely to have affected the outcome), but clusters were analysed in their original group of assignment and randomisation | Participants and people delivering the interventions were aware of intervention groups during the trial and there were reported deviations from intended intervention that arose because of trial context (and may are likely to have affected the outcome), but clusters were analysed in their original group of assignment and randomisation |
|  | Optional: What is the predicted direction of bias due to deviations from intended interventions? | Nil | Nil | Nil | Nil | Nil | Nil | Nil |
|  | Description/Support for judgement | Nil | Nil | Nil | Nil | Nil | Nil | Nil |
| Bias due to missing outcome data | 3.1a Were outcome data available for all, or nearly all, clusters randomized? | Y | Y | Y | Y | Y | Y | Y |
|  | Description/Support for judgement | Quote: "The total number of participants analyzed was 1297 with 367 in the face mask and hand hygiene group (9 deemed ineligible and 26 lost to follow-up), 378 in the face mask–only group (11 deemed ineligible and 52 lost to follow-up), and 552 in the control group (19 deemed ineligible and 21 lost to follow-up) (Figure A1 in the Appendix). In total, 1297 (97%) of 1331 participants completed a baseline and at least 1 weekly survey", Table 1 Comment: All 7 residence halls randomised provided outcome data | Quote: Figure 1: 23/465 randomised households were lost to follow-up and data was not available for these households, "Across all study arms, 343 (29.9%) of 1147 family members in the 442 households", "One hundred and thirteen infections in 94 households were coindex cases identified on the Day 1 home visit. We excluded these 94 households from the analysis, because the true index case could not be established and these infections had occurred before interventions had been implemented. Therefore, the intent to treat analysis included 348 households and 885 members." | Quote: Table S5, Table S1, Figure 1 Comment: Data from all 37 residence houses randomised were available | Quote: Table S5, Table S1, Figure 1 Comment: Data from all 37 residence houses randomised were available | Figure 1, Table 1,Table 2 | Figure 1, Table 1, Table 2 | Quote: Table 1 |
|  | 3.1b Were outcome data available for all, or nearly all, participants within clusters? | Y | Y | Y | Y | Y | Y | Y |
|  | Description/Support for judgement | Quote: "The total number of participants analyzed was 1297 with 367 in the face mask and hand hygiene group (9 deemed ineligible and 26 lost to follow-up), 378 in the face mask–only group (11 deemed ineligible and 52 lost to follow-up), and 552 in the control group (19 deemed ineligible and 21 lost to follow-up) (Figure A1 in the Appendix). In total, 1297 (97%) of 1331 participants completed a baseline and at least 1 weekly survey", Table 1 Comment: Outcome data was obtained from all subjects randomised, except those ineligible for inclusion or were lost to follow up (completion were 88-92%) | Quote: Figure 1: 23/465 randomised households were lost to follow-up and data was not available for these households, "Across all study arms, 343 (29.9%) of 1147 family members in the 442 households", "One hundred and thirteen infections in 94 households were coindex cases identified on the Day 1 home visit. We excluded these 94 households from the analysis, because the true index case could not be established and these infections had occurred before interventions had been implemented. Therefore, the intent to treat analysis included 348 households and 885 members." | Quote: Table S5, Table S1, Figure 1 Comment: Data from all 1,111 of the 1188 participants randomised were available | Quote: Table S5, Table S1, Figure 1 Comment: Data from all 1,111 of the 1188 participants randomised were available | Quote: Figure 1, Table 1,Table 2 | Quote: Figure 1, Table 1,Table 2 | Quote: Tables 1 & 2 Comment: Study did not provide breakdown of outcomes in control group according to compliance |
|  | 3.2 If N/PN/NI to 3.1a or 3.1b: Are the proportions of missing outcome data and reasons for missing outcome data similar across intervention groups? | N/A | N/A | N/A | N/A | N/A | N/A | N/A |
|  | Description/Support for judgement | Nil | Nil | Nil | Nil | Nil | Nil | Nil |
|  | 3.3 If N/PN/NI to 3.1a or 3.1b: Is there evidence that results were robust to the presence of missing outcome data? | N/A | N/A | N/A | N/A | N/A | N/A | N/A |
|  | Description/Support for judgement | Nil | Nil | Nil | Nil | Nil | Nil | Nil |
|  | **Risk of bias judgement** | **Low** | **Low** | **Low** | **Low** | **Low** | **Low** | **Low** |
|  | General Note | Observed number of events is much higher than the number of participants with missing outcome data, hence bias will necessarily be small as outcome data were available for nearly all randomised participants | Observed number of events is much higher than the number of participants with missing outcome data, hence bias will necessarily be small as outcome data were available for nearly all randomised participants | Observed number of events is much higher than the number of participants with missing outcome data, hence bias will necessarily be small as outcome data were available for nearly all randomised participants | Observed number of events is much higher than the number of participants with missing outcome data, hence bias will necessarily be small as outcome data were available for nearly all randomised participants | Observed number of events is much higher than the number of participants with missing outcome data, hence bias will necessarily be small as outcome data were available for nearly all randomised participants | Observed number of events is much higher than the number of participants with missing outcome data, hence bias will necessarily be small as outcome data were available for nearly all randomised participants | Observed number of events is much higher than the number of participants with missing outcome data, hence bias will necessarily be small as outcome data were available for nearly all randomised participants |
|  | Optional: What is the predicted direction of bias due to missing outcome data? | Nil | Nil | Nil | Nil | Nil | Nil | Nil |
|  | Description/Support for judgement | Nil | Nil | Nil | Nil | Nil | Nil | Nil |
| Bias in measurement of the outcome | 4.1a Were outcome assessors aware that a trial was taking place? | PY | NI | PY | Y | NI | PY | PY |
|  | Description/Support for judgement | Quote: "Written informed consent was obtained from all participants.", "Participants were also asked to complete the baseline and weekly Web-based surveys concerning the occurrence of respiratory illness symptoms and the use of interventions during the study", "During scheduled participant visits, study nurses ascertained date of illness onset, temperature, use of antipyretics, and reported symptoms (cough, feverishness, chills, body aches, headache, nasal congestion, and sore throat)." Comment: outcome assessors were participants themselves and study nurses; awareness of the trail is highly possible since informed consent was obtained, but we do not have access to recruitment materials used to determine if the subjects were briefed of other possible interventions, or just their intervention since the clusters were likely randomised prior to recruitment. Nonetheless the US regulations for human subjects states that informed consent includes "a description of the procedures to be followed, and identification of any procedures which are experimental;" | Quote: "Laboratory methods" section did not mention whether the tests were outsourced to an external lab or done by study staff, and whether the staff conducting the tests were blinded | Quote: "All study participants were given materials describing the ILI case definition (presence of cough and at least one or more of fever/feverishness, chills, or body aches) and contact information of clinical research staff for illness assessment. Clinical research staff recorded the date of illness onset, body temperature, use of anti-pyretics, and reported symptoms. Clinical research staff recorded the date of illness onset, body temperature, use of anti-pyretics, and reported symptoms. Throat swab specimens were tested for influenza A or B using real-time polymerase chain reaction (Rt-PCR)." Comment: Publication also did not mention who will conduct/assess the laboratory test but it is possible that they are part of the research team and hence, aware of the trial taking place | Quote: All study participants were given materials describing the ILI case definition (presence of cough and at least one or more of fever/feverishness, chills, or body aches) and contact information of clinical research staff for illness assessment. Clinical research staff recorded the date of illness onset, body temperature, use of anti-pyretics, and reported symptoms.  Comment: Outcome assessors were participants themselves and study nurses; awareness of the trial is highly possible since informed consent was obtained, but we do not have access to recruitment materials used to determine if the subjects were briefed of other possible interventions, or just their intervention since the clusters were likely randomised prior to recruitment. Nonetheless the US regulations for human subjects states that informed consent includes "a description of the procedures to be followed, and identification of any procedures which are experimental;" | Quote: "Specimens were analysed by qRT-PCR at the Centre for Biological Security, Division of Highly-Pathogenic Viruses (season 2009/10) and the National Reference Centre for Influenza (season 2010/11) both part of the Robert Koch Institute in Berlin, Germany." Comment: Did not mention if the laboratory technician were aware of the trial however, they were from the same institute as the investigators | Quote: "All participants self-recorded symptoms (fever, shivering, measured temperature, cough, sore throat) and daily routines (incl. the time spent at home, and within close range (i.e. < 2 m) of the index patient) in a daily monitoring questionnaire. A secondary outcome measure was the occurrence of ILI as defined by WHO [11] as fever plus cough or sore throat." Comment: Secondary outcome was participant reported and hence it is possible since informed consent was obtained, but we do not have access to recruitment materials used to determine if the subjects were briefed of other possible interventions during consent taking, or just their intervention since the clusters were likely randomised prior to recruitment. Nonetheless the US regulations for human subjects states that informed consent includes "a description of the procedures to be followed, and identification of any procedures which are experimental" It is likely that they are aware that they are part of a trial | Quote: "Subjects reporting ILI symptoms and their close tent contacts were followed for up to 5 days from the date of recruitment into the study using their diary and text message/ telephonic reminders. They were reminded by phone and/or email to return their completed diaries after Hajj with clear information on the progress of their ILI." Comment: Symptoms were self-reported and hence participants were outcome assessors. It is possible the participants knew they were in a trial since informed consent was obtained, but we do not have access to recruitment materials used to determine if the subjects were briefed of other possible interventions during consent taking, or just their intervention since the clusters were likely randomised prior to recruitment. Nonetheless the Australian regulations for human subjects states that informed consent includes "An information and consent document or other consent strategy should be appropriate to the needs of the participants and proportional to the project’s risks and ethical sensitivity (Section 3.1.3)" It is likely that they are aware that they are part of a trial; however it is unknown whether lab personnel conducting test knew of the allocation |
|  | 4.1b If Y/PY/NI to 4.1: Were outcome assessors aware of the intervention received by study participants? | PY | NI | PY | Y | NI | PY | PY |
|  | Description/Support for judgement | Quote: "All participants received basic hand hygiene education (proper hand hygiene practices and cough etiquette) through an email video link and the study Web site. In addition, face mask and hand hygiene group participants received written materials detailing appropriate hand sanitizer and mask use; ..(all the way till)... Mask and hand hygiene group participants also received alcohol-based hand sanitizer (portable 2 oz squeeze bottle; 8 oz pump) for use throughout the study", " Because of the inability to blind participants to study interventions, compliance with these interventions must be considered carefully" Comment: outcome assessors were participants themselves and study nurses; nature of intervention made it impossible to blind participants to their intervention but it was unclear whether study nurses were aware of the intervention received by the participants and hence, a "PY" instead of "Y" was assigned | Quote: "Laboratory methods" section did not mention whether the tests were outsourced to an external lab or done by study staff, and whether the staff conducting the tests were blinded; other outcomes were self-reported by participants | Quote: "All study participants were given materials describing the ILI case definition (presence of cough and at least one or more of fever/feverishness, chills, or body aches) and contact information of clinical research staff for illness assessment. Clinical research staff recorded the date of illness onset, body temperature, use of anti-pyretics, and reported symptoms. Clinical research staff recorded the date of illness onset, body temperature, use of anti-pyretics, and reported symptoms. Throat swab specimens were tested for influenza A or B using real-time polymerase chain reaction (Rt-PCR)." Comment: Publication also did not mention who will conduct/assess the laboratory test but it is possible that they are part of the research team and hence, aware of the trial taking place | Quote: All study participants were given materials describing the ILI case definition (presence of cough and at least one or more of fever/feverishness, chills, or body aches) and contact information of clinical research staff for illness assessment. Clinical research staff recorded the date of illness onset, body temperature, use of anti-pyretics, and reported symptoms.  Comment: Outcome assessors were participants themselves and study nurses; awareness of the trial is highly possible since informed consent was obtained, but we do not have access to recruitment materials used to determine if the subjects were briefed of other possible interventions, or just their intervention since the clusters were likely randomised prior to recruitment. Nonetheless the US regulations for human subjects states that informed consent includes "a description of the procedures to be followed, and identification of any procedures which are experimental;" | Quote: "Specimens were analysed by qRT-PCR at the Centre for Biological Security, Division of Highly-Pathogenic Viruses (season 2009/10) and the National Reference Centre for Influenza (season 2010/11) both part of the Robert Koch Institute in Berlin, Germany." Comment: Did not mention if the laboratory technician were aware of the trial however, they were from the same institute as the investigators | Quote: "All participants self-recorded symptoms (fever, shivering, measured temperature, cough, sore throat) and daily routines (incl. the time spent at home, and within close range (i.e. < 2 m) of the index patient) in a daily monitoring questionnaire. A secondary outcome measure was the occurrence of ILI as defined by WHO [11] as fever plus cough or sore throat." Comment: Secondary outcome was participant reported and hence it is possible since informed consent was obtained, but we do not have access to recruitment materials used to determine if the subjects were briefed of other possible interventions during consent taking, or just their intervention since the clusters were likely randomised prior to recruitment. Nonetheless the US regulations for human subjects states that informed consent includes "a description of the procedures to be followed, and identification of any procedures which are experimental" It is likely that they are aware that they are part of a trial | Quote: "Subjects reporting ILI symptoms and their close tent contacts were followed for up to 5 days from the date of recruitment into the study using their diary and text message/ telephonic reminders. They were reminded by phone and/or email to return their completed diaries after Hajj with clear information on the progress of their ILI.", "Two nasal swabs, one from each nostril, were taken from each case of ILI by trained collectors at the time of recruitment and from all symptomatic contacts within 2-3 days of symptom onset." Comemnt: As all pilgrims were encouraged to wear a mask during the hajj, it is unclear if the outcome assessors (the trained collectors) could distinguish which tent was the intervention arm or if they knew beforehand. However, ILI symptoms were patient reported and it was likely that participants in the intervention group knew of their assignment since informed consent was obtained, but we do not have access to recruitment materials used to determine if the subjects were briefed of other possible interventions during consent taking, or just their intervention since the clusters were likely randomised prior to recruitment. Nonetheless the Australian regulations for human subjects states that informed consent includes "An information and consent document or other consent strategy should be appropriate to the needs of the participants and proportional to the project’s risks and ethical sensitivity (Section 3.1.3)" It is likely that they are aware that they are part of a trial |
|  | 4.2 If Y/PY/NI to 4.1: Was the assessment of the outcome likely to be influenced by knowledge of intervention received? | NI | N | N | NI | N | NI | PY |
|  | Description/Support for judgement | Quote: "The weekly surveys included questions regarding ILI symptoms, intervention compliance, and health and hygiene behaviors...All residents in participating halls received promotional materials describing the ILI case definition (presence of cough and at least 1 constitutional symptom [fever/feverishness, chills, or body aches]) [7] and phone numbers for contacting the nursing staff to assess for ILI symptoms. During scheduled participant visits, study nurses ascertained date of illness onset, temperature, use of antipyretics, and reported symptoms (cough, feverishness, chills, body aches, headache, nasal congestion, and sore throat)" Comment: ILI episodes were reported using symptoms and had a certified medical worker (study nurse) to assess the symptoms. However, as the outcome is a combination of partcipant-reported outcomes and observer reported outcomes involving some judgement, more information regarding the awareness of the intervention received by the study nurse is required to assess whether the assessment by the study nurse is likely influenced by their knowledge of the intervention received. | Quote: "Specimens were aliquoted and tested by qualitative rRT-PCR to detect influenza viral RNA. Blood specimens were collected from each consenting household member on Day 0 ⁄ 1 and again on Day 21 for serological testing by hemagglutinin inhibition (HI) assay to identify asymptomatic infection and correlate with qualitative PCR results. A fourfold rise in HI antibody titer in paired sera was considered to be evidence of an acute influenza virus infection."  Comment: Outcome reported by observer does not require judgement | Quote: "Positive samples were identified using PCR samples tested using the TaqMan System (Applied Biosystem, Foster City, CA, USA). Primers and probes were developed by the CDC Influenza Branch to detect influenza types A and B as previously noted" Comment: Outcome reported by observer does not require judgement | Quote: "Participants were also asked to complete on-line weekly surveys and to report the presence/absence of illness symptoms. Weekly surveys included questions on ILI symptoms, intervention compliance", "All study participants were given materials describing the ILI case definition (presence of cough and at least one or more of fever/feverishness, chills, or body aches) and contact information of clinical research staff for illness assessment. Clinical research staff recorded the date of illness onset, body temperature, use of anti-pyretics, and reported symptoms" Comment: ILI episodes were reported using symptoms and had a clinical research staff to assess the symptoms. However, as the outcome is a combination of partcipant-reported outcomes and observer reported outcomes involving some judgement, more information regarding the awareness of the intervention received by the clinical research staff is required to assess whether the assessment by the staff is likely influenced by their knowledge of the intervention received. | Quote: "The primary outcome measure for secondary cases was qRT-PCR confirmed influenza infection." Comment: Outcome reported by observer does not require judgement The outcomes are based on the laboratory resultThe outcomes are based on the laboratory result | Quote: "A secondary outcome measure was the occurrence of ILI as defined by WHO [11] as fever plus cough or sore throat" Comment: ILI episodes were reported using symptoms and had a certified medical worker (study nurse) to assess the symptoms. However, as the outcome is a combination of partcipant-reported outcomes and observer reported outcomes involving some judgement, more information regarding the awareness of the intervention received by the study nurse is required to assess whether the assessment by the study nurse is likely influenced by their knowledge of the intervention received. | Quote: "Subjects reporting ILI symptoms and their close tent contacts were followed for up to 5 days from the date of recruitment into the study using their diary and text message/ telephonic reminders. They were reminded by phone and/or email to return their completed diaries after Hajj with clear information on the progress of their ILI.", "Two nasal swabs, one from each nostril, were taken from each case of ILI by trained collectors at the time of recruitment and from all symptomatic contacts within 2-3 days of symptom onset. One swab was used for influenza point of care testing (POCT) using the QuickVue Influenza (A+B) assay (Quidel Corporation, San Diego, USA), and the other was immersed in universal viral transport media for later nucleic acid testing (NAT)"  Comment: ILI episodes were reported using questionnaire and symptoms diary and hwere also tested with lab test. However, although the outcome is a combination of partcipant-reported outcomes and observer reported outcomes not requiring judgement, the primary results reported was participant-reported and could be influenced by their knowledge of the intervention received. |
|  | **Risk of bias judgement** | **Some concerns** | **Low** | **Low** | **Some Concern** | **Low** | **Some concerns** | **High** |
|  | General Note | Insufficient information was provided on whether all outcome assessors were aware of the trial taking place and the intervention received by study participants, although it is highly likely. As the assessment of the outcome was subjective (patient reported and judgement required from observer), it could be influenced by knowledge of the intervntion received and more information is required to assess the level of bias from measurement of outcome | Insufficient information was provided on whether outcome assessors were aware of the trial taking place and the intervention received by study participants, but the assessment of the outcome was objective and unlikely to been influenced by knowledge of the intervntion received | Insufficient information was provided on whether outcome assessors were aware of the trial taking place and the intervention received by study participants, but the assessment of the outcome was objective and unlikely to been influenced by knowledge of the intervntion received | Insufficient information was provided on whether all outcome assessors were aware of the trial taking place and the intervention received by study participants, although it is highly likely. As the assessment of the outcome was subjective (patient reported and judgement required from observer), it could be influenced by knowledge of the intervntion received and more information is required to assess the level of bias from measurement of outcome | Insufficient information was provided on whether all outcome assessors were aware of the trial taking place and the intervention received by study participants, although it is highly likely. As the assessment of the outcome was subjective (patient reported and judgement required from observer), it could be influenced by knowledge of the intervntion received and more information is required to assess the level of bias from measurement of outcome | Insufficient information was provided on whether all outcome assessors were aware of the trial taking place and the intervention received by study participants, although it is highly likely. As the assessment of the outcome was subjective (patient reported and judgement required from observer), it could be influenced by knowledge of the intervntion received and more information is required to assess the level of bias from measurement of outcome | Nil |
|  | Optional: What is the predicted direction of bias due to measurement of the outcome? | Nil | Nil | Nil | Nil | Nil | Nil | Nil |
|  | Description/Support for judgement | Nil | Nil | Nil | Nil | Nil | Nil | Nil |
| Bias in selection of the reported result | 5.1.Are the reported outcome data likely to have been selected, on the basis of the results, from multiple outcome measurements (e.g. scales, definitions, time points) within the outcome domain? | PY | PN | PN | PY | PN | PN | PN |
|  | Description/Support for judgement | Quote: "The weekly surveys included questions regarding ILI symptoms, intervention compliance, and health and hygiene behaviors...All residents in participating halls received promotional materials describing the ILI case definition (presence of cough and at least 1 constitutional symptom [fever/feverishness, chills, or body aches]) [7] and phone numbers for contacting the nursing staff to assess for ILI symptoms. During scheduled participant visits, study nurses ascertained date of illness onset, temperature, use of antipyretics, and reported symptoms (cough, feverishness, chills, body aches, headache, nasal congestion, and sore throat).Students with ILI were offered $25.00 for providing a throat specimen. With a cotton swab, nurses collected specimens and transferred them to veal infusion broth. Samples were processed and analyzed using standard laboratory methods as described in Section A1 of the Appendix"; "The main outcome variable was the first reported ILI that was based on clinical ascertainment or survey report (if no available clinical report) over the 6-week study period. A small number of cases reported >1 ILI (15 cases); only the first ILI was included in our analyses." Comment: Outcome was reported as HR/ time to first ILI episode, although the outcome was reported >1 times in a few subjects without reason for excluding them. hence there is evidence to suspect that the reported numerical results could have been selected | Quote: "During each subsequent home visit, one nasal swab and one throat swab were collected from the index case and from all household contacts...Approximately 5 ml of serum collected at Days 1 and 21 were tested for antibody seroconversion using the WHO Haemagglutinin Inhibition kit (provided by US CDC Atlanta) per manufacturers’ recommendations using 0Æ75% guinea pig red blood cells resuspended in PBS and BSA","A secondary influenza virus infection was defined as a positive rRT-PCR result on Days 3 or 7 or a fourfold rise in influenza HI antibody titers with the virus type and subtype matching the index case." Comment: Secondary influenza episode measured using laboratory tests, which was described in details | Quote: "Positive samples were identified using PCR samples tested using the TaqMan System (Applied Biosystem, Foster City, CA, USA). Primers and probes were developed by the CDC Influenza Branch to detect influenza types A and B as previously noted" Comment: Positive samples were identified using PCR whenever there was a self-reported ILI episode, and there is no oportunity to select from multiple measres. | Participants were also asked to complete on-line weekly surveys and to report the presence/absence of illness symptoms. Weekly surveys included questions on ILI symptoms, intervention compliance (e.g. total mask hours per day and frequency of alcohol-based hand sanitizer use), and health and hand hygiene practices.   Clinical research staff recorded the date of illness onset, body temperature, use of anti-pyretics, and reported symptoms. Comment: Outcome was reported as HR/ time to first ILI episode, although the outcome was reported >1 times in a few subjects without reason for excluding them. hence there is evidence to suspect that the reported numerical results could have been selected | Quote: "The observation period for each household lasted 8 days, starting on the day of symptom onset of the index patient (day 1) we obtained nasal wash specimens (or - if these were not possible - nasal swabs) from all participating household members.", "We defined a symptomatic secondary influenza virus infection as a laboratory confirmed influenza infection in a household member who developed fever (> 38.0°C), cough, or sore-throat during the observation period."  Comment: Secondary influenza episode measured using laboratory tests, which was described in details. only first episode was reported despite multiple measurements taken during follow-up period. however, follow-up period was too short for a contact to develop more than 1 episode and hence the reported outcome data is unlikely to have been selected from multiple outcome measurements | Quote: "The observation period for each household lasted 8 days, starting on the day of symptom onset of the index patient (day 1) we obtained nasal wash specimens (or - if these were not possible - nasal swabs) from all participating household members.", "We termed all other secondary cases as subclinical. A secondary outcome measure was the occurrence of ILI as defined by WHO [11] as fever plus cough or sore throat."  Comment: ILI was defined using WHO definition, which was described in details. only first episode was reported despite multiple measurements taken during follow-up period. however, follow-up period was too short for a contact to develop more than 1 episode and hence the reported outcome data is unlikely to have been selected from multiple outcome measurement | Only secondary influenza was assessed at time point mentioned. |
|  | 5.2 Are the reported outcome data likely to have been selected, on the basis of the results, from multiple analyses of the data? | PN | PN | PN | PN | PN | PN | PY |
|  | Description/Support for judgement | Quote: "Statistical analyses" and "Survival analyses" section Comment: There is clear evidence that all eligible reported results for the outcome measurement correspond to all intended analyses | Quote:"Statistical analyses" section, Tables 2-4  Comment: There is clear evidence that all eligible reported results for the outcome measurement correspond to all intended analyses | Quote: "Statistical analyses" Comment: The reported outcome result follows the intended protocol | Quote: "Statistical analyses" Comment: The reported outcome result follows the intended protocol | Quote: "Statistical analyses" Comment: The reported outcome result follows the intended protocol | Quote: "Statistical analyses" Comment: The reported outcome result follows the intended protocol | Quote: Table 2 Comment: Did not provide breakdown of outcome in control group according to compliance even though a significant proportion deviated (by study standards) and wore masks, compliance was also not pre-specified in methods. |
|  | **Risk of bias judgement** | **High** | **Low** | **Low** | **High** | **Low** | **Low** | **High** |
|  | General Note | The result being assessed is unlikely to have been selected on the basis of the results, from multiple eligible analyses of the data but may have been selected from multiple eligible outcome measurements within the outcome domain, based on the multiple outcone measurements taken at different time points but only one being presented (first ILI episode only) | The result being assessed is unlikely to have been selected on the basis of the results, from multiple eligible outcome measurements within the outcome domain, and multiple eligible analyses of the data | The result being assessed is unlikely to have been selected on the basis of the results, from multiple eligible outcome measurements within the outcome domain, and multiple eligible analyses of the data | The result being assessed is unlikely to have been selected on the basis of the results, from multiple eligible analyses of the data but may have been selected from multiple eligible outcome measurements within the outcome domain, based on the multiple outcone measurements taken at different time points but only one being presented (first ILI episode only) | The result being assessed is unlikely to have been selected on the basis of the results, from multiple eligible outcome measurements within the outcome domain, and multiple eligible analyses of the data | The result being assessed is unlikely to have been selected on the basis of the results, from multiple eligible outcome measurements within the outcome domain, and multiple eligible analyses of the data | Nil |
|  | Optional: What is the predicted direction of bias due to selection of the reported result? | Nil | Nil | Nil | Nil | Nil | Nil | Nil |
|  | Description/Support for judgement | Nil | Nil | Nil | Nil | Nil | Nil | Nil |
| Summary of bias in all doamains | Risk of bias judgement (Randomisation) | High | High | **High** | **High** | **High** | **High** | **High** |
|  | Risk of bias judgement (timing of identification and recruitment of individual participants in relation to timing of randomization) | High | Low | High | High | Low | Low | Low |
|  | Risk of bias judgement (deviations from intended interventions) | Some concerns | Low | Low | Low | High | High | Some Concerns |
|  | Risk of bias judgement (missing outcome data) | Low | Low | Low | Low | Low | Low | Low |
|  | Risk of bias judgement (measurement of outcome data) | Some concerns | Low | Low | Some Concern | Low | Some concerns | High |
|  | Risk of bias judgement (Selection of the reported result) | High | Low | Low | High | Low | Low | High |
| Overall bias | **Risk of bias judgement (overall)** | **High** | **High** | **High** | **High** | **High** | **High** | **High** |
|  | General Note | Nil | Nil | Nil | Nil | Nil | Nil | Nil |
|  | Optional: What is the predicted direction of bias due to selection of the reported result? | Nil | Nil | Nil | Nil | Nil | Nil | Nil |
|  | Description/Support for judgement | Nil | Nil | Nil | Nil | Nil | Nil | Nil |

### Table S6. Breakdown of reporting quality assessment ratings for each included randomised controlled trial

| Author,  Year  Question | Aiello, 2010 | Simmerman, 2011 | Aiello, 2012 | Suess, 2012 | Barasheed, 2014 |
| --- | --- | --- | --- | --- | --- |
| 1a | N | N | N | Y | N |
| 1b | P | P | P | P | Y |
| 2a | P | P | P | P | P |
| 2b | Y | Y | Y | Y | Y |
| Reporting quality for Title, Abstract and Introduction | Fair | Fair | Fair | Fair | High |
| 3a | Y | Y | P | Y | Y |
| 3b | N/A | N/A | N/A | N/A | N |
| 4a | Y | N | N | Y | P |
| 4b | Y | Y | Y | Y | Y |
| 5 | Y | Y | Y | Y | Y |
| 6a | Y | Y | Y | Y | Y |
| 6b | N/A | N/A | N/A | N/A | N |
| 7a | Y | N | Y | Y | N |
| 7b | N/A | N/A | N/A | N/A | N |
| 8a | Y | Y | P | P | N |
| 8b | Y | Y | Y | Y | P |
| 9 | Y | Y | P | Y | N |
| 10a | N | P | P | Y | P |
| 10b | Y | Y | P | P | Y |
| 10c | Y | Y | Y | Y | Y |
| 11a | P | P | P | Y | Y |
| 11b | N/A | N/A | N/A | N/A | N/A |
| 12a | Y | Y | Y | Y | P |
| 12b | Y | Y | Y | Y | N |
| Reporting quality for Methods | High | High | High | High | High |
| 13a | P | Y | Y | Y | P |
| 13b | P | Y | Y | Y | P |
| 14a | Y | Y | Y | Y | N |
| 14b | P | N | P | P | Y |
| 15 | N | Y | P | P | N |
| 16 | P | Y | Y | Y | P |
| 17a | P | P | Y | Y | N |
| 17b | P | Y | P | Y | N |
| 18 | Y | Y | Y | Y | P |
| 19 | N/A | N/A | N/A | N/A | N |
| Reporting quality for Results | Fair | High | High | High | Low |
| 20 | Y | Y | Y | Y | N |
| 21 | Y | Y | Y | Y | Y |
| 22 | Y | Y | Y | Y | Y |
| Reporting quality for Discussion | High | High | High | High | High |
| 23 | Y | N | Y | Y | Y |
| 24 | N | N | Y | N | N |
| 25 | Y | P | Y | Y | P |
| Reporting quality for Other information | High | Low | High | High | Low |
| Overall reporting quality (Using qualities for methods, results & discussion) | High | High | High | High | Fair |
| Overall reporting quality (Using qualities for title, abstract & introduction, methods, results & discussion) | High | High | High | High | Fair |

### Table S7. Breakdown of methodological and reporting quality assessment ratings for each included observational study

|  | Study Design | Cohort & Cross sectional Studies | | | | | | | | Case-Control | |
| --- | --- | --- | --- | --- | --- | --- | --- | --- | --- | --- | --- |
| Methodology Quality^1^ | Author,  Year  Question | Deris, 2010 | Gautret, 2011 | Al-Jasser, 2012 | Balaban, 2012 | Kim, 2012 | Gautret, 2015 | Hashim, 2016 | Uchida, 2017 | Emamian, 2013 | Zhang, 2013 |
|  | 1 | Y | P | Y | Y | Y | P | Y | P | Y | P |
|  | 2 | P | P | Y | P | Y | Y | Y | Y | P | Y |
|  | 3 | Y | CT | CT | CT | N | CT | N | Y | N | N |
|  | 4 | CT | CT | Y | P | Y | Y | Y | Y | Y | Y |
|  | 5 | P | N | Y | N | N | N | P | N | Y | P |
|  | 6 | N | P | N | N | N | N | N | N | Y | Y |
|  | 7 | Y | Y | Y | Y | N | CT | N | N | P | N |
|  | 8 | N | P | Y | N | Y | N | N | N | Y | N |
|  | 9 | N | P | P | P | P | N | N | N | N | N |
|  | 10 | N | N | N | N | N | N | N | N | P | P |
|  | 11 | N | N | P | P | Y | P | N | N | Y | N |
|  | 12 | N | N | N | N | P | N | N | N | P | N |
|  | 13 | N/A | N | Y | Y | N/A | CT | N/A | N/A |  |  |
|  | 14 | P | N | N | Y | P | N | Y | Y |  |  |
|  | Selection | Fair | Fair | High | Fair | High | High | High | High | High | High |
|  | Misclassification | Low | Fair | Low | Low | Low | Low | Low | Low | Low | Low |
|  | Detection | Low | Low | Low | Low | Fair | Low | Low | Low | High | Low |
|  | Confounding | Fair | Low | Low | High | Fair | Low | High | High | High | High |
|  | Other (Inappropriate sample size, attrition) | Fair | Low | High | Low | Low | Low | Fair | Low | Low | Low |
| Reporting Quality^2^ | 1a | Y | P | Y | N | N | N | Y | P | Y | P |
|  | 1b | Y | P | P | P | P | Y | Y | Y | Y | P |
|  | 2 | Y | P | Y | Y | Y | Y | Y | Y | Y | P |
|  | 3 | Y | P | Y | Y | Y | Y | Y | P | Y | P |
|  | Reporting quality for Title, Abstract and Introduction | High | Fair | High | High | High | High | High | High | High | Fair |
|  | 4 | Y | Y | Y | Y | Y | Y | Y | P | Y | Y |
|  | 5 | P | Y | Y | Y | Y | Y | Y | Y | P | Y |
|  | 6a | P | P | Y | P | Y | Y | Y | Y | Y | P |
|  | 6b | N/A | N | N/A | N/A | N/A | N/A | N/A | N/A | Y | N/A |
|  | 7 | P | Y | P | Y | P | Y | P | P | P | P |
|  | 8 | P | P | P | P | Y | Y | Y | Y | P | P |
|  | 9 | N | N | N | N | N | N | N | N | N | N |
|  | 10 | Y | P | Y | P | N | Y | Y | N | N | Y |
|  | 11 | P | N | N | P | Y | N | N | N | P | N |
|  | 12a | Y | P | Y | P | Y | Y | Y | Y | Y | Y |
|  | 12b | N | Y | Y | Y | N | N | Y | Y | N | N |
|  | 12c | P | Y | Y | Y | Y | N | Y | Y | N | P |
|  | 12d | N/A | N | N | Y | N/A | N | N | N | N | N |
|  | 12e | N | N | N | N | N | N | N | N | N | N |
|  | Reporting quality for Methods | Fair | Low | High | High | High | High | High | High | Low | Low |
|  | 13a | P | Y | P | P | Y | P | Y | Y | Y | Y |
|  | 13b | N/A | N | N | P | Y | N | N | N | N | Y |
|  | 13c | N | N | N | N | Y | N | N | N | N | N |
|  | 14a | P | Y | Y | Y | Y | Y | Y | Y | Y | P |
|  | 14b | P | Y | Y | Y | Y | N | Y | Y | N | Y |
|  | 14c | N/A | P | N/A | N/A | N/A | N/A | N/A | N/A | N/A | N/A |
|  | 15 | Y | Y | Y | Y | Y | Y | Y | Y | Y | Y |
|  | 16a | Y | P | Y | Y | P | N | Y | Y | Y | Y |
|  | 16b | Y | Y | Y | Y | N | Y | Y | NA | Y | Y |
|  | 16c | N | N | N | N | N | N | N | N | N | N |
|  | 17 | N | N | N | Y | N | N | Y | P | N | N |
|  | Reporting quality for Results | Low | High | High | High | High | Low | High | High | Low | High |
|  | 18 | Y | Y | Y | Y | P | Y | P | Y | Y | P |
|  | 19 | P | P | P | P | P | P | P | P | P | P |
|  | 20 | Y | P | Y | Y | Y | P | Y | Y | P | Y |
|  | 21 | N | Y | N | Y | N | Y | N | Y | N | N |
|  | Reporting quality for Discussion | High | Fair | High | High | Fair | Fair | Fair | High | Fair | Fair |
|  | 22 | P | N | Y | N | N | N | P | P | N | N |
|  | Reporting quality for Other information | Fair | Low | High | Low | Low | Low | Fair | Fair | Low | Low |
|  | Overall reporting quality (Using qualities for methods, results & discussion) | Low | Low | High | High | High | Low | High | High | Low | Low |
|  | Overall reporting quality (Using qualities for title, abstract & introduction, methods, results & discussion) | Fair | Low | High | High | High | Fair | High | High | Low | Low |

Y, Yes; N, No; P, Partially reported/done; CT, Can’t tell; N/A, Not Applicable

# Appendix C

### Figure S1. Conventional funnel plot of studies included in meta-analysis (n=10 studies), grouped by age group


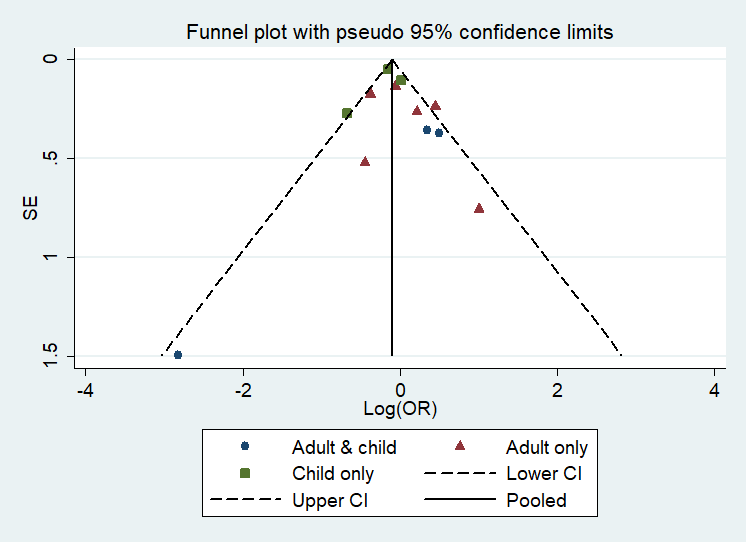


### Figure S2. Contoured funnel plot of studies included in meta-analysis (n=10 studies)


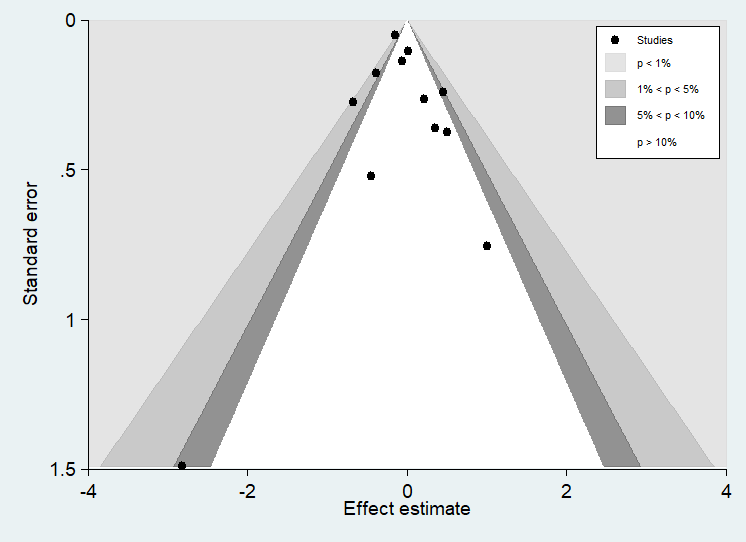


# Appendix D

### Table S8. Summary of Findings in additional intervention arm (Face mask + hand hygiene)

| **Author, Year [Ref.]** | **Population size (% Men); Percentage with recent flu vaccine in alternative intervention arm** | **Description of measures implemented in alternative intervention arm** | **Reported summary risk estimate: Risk Ratio [RR], Odds Ratio [OR], or Hazard Ratio [HR] (95% Confidence Interval);**  **% Population infected in intervention arm (infected/population size)** | **Key Findings** |
| --- | --- | --- | --- | --- |
| Aiello (2010) | 367 (51.2%); 12.8% | Wearing surgical facemask as much as possible in residence hall during intervention + use of alcohol-based hand sanitiser | Adjusted HR^1^: 0.87 (0.73-1.02);  25.1% (92/367) | - **Significantly decreased ILI incidence rate** (P<0.025) in face mask and hand hygiene group, compared to control group from 4^th^ week of intervention onwards - Adjusted rate ratio (95% CI):   4^th^ week: 0.65 (0.47-0.91)  5^th^ week: 0.56 (0.36-0.88)  6^th^ week: 0.49 (0.27-0.87) |
| Aiello (2012) | 347 (48.4%); 15.9% | Wearing surgical facemask as much as possible in residence hall during intervention  + use of alcohol-based hand sanitiser | Self-reported/clinically diagnosed ILI: adjusted HR^2^: 0.78 (0.57-1.08);  8.9% (31/347)  Laboratory-confirmed influenza:  Adjusted HR^2^: 0.57 (0.26-1.24);  1.7% (6/347) | - **Significantly decreased ILI incidence rate** (P<0.05) in face mask and hand hygiene group, compared to control group from 3^rd^ week of intervention onwards - Adjusted rate ratio (95% CI):   3^rd^ week: 0.52 (0.30-0.88)  4^th^ week: 0.40 (0.20-0.83)  5^th^ week: 0.32 (0.12-0.84)  6^th^ week: 0.25 (0.07-0.87)   - No significant reduction in laboratory-confirmed influenza incidence |
| Suess (2012) | 67 (49.3%); 3.0% | Healthy household members to wear masks at all times when in one room with the index  patient and/or any other household member with  respiratory symptoms + use of alcohol-based hand-rub | Self-reported/clinically diagnosed ILI: adjusted OR^3^: 0.5 (0.16-1.58);  17.6%% (6/34)  Laboratory-confirmed influenza:  Adjusted OR^3^: 0.62 (0.23-1.65);  30.3% (10/33) | - No significant difference in secondary attack rate across the groups, regardless whether the case was defined with ILI or laboratory-confirmed influenza definition, and after stratification for influenza season, virus subtype or timing of the first household visit. - No significant difference in odds of laboratory-confirmed influenza incidence **i**n mask & hand hygiene group when per-protocol analysis was utilised. - Control OR (95% CI): Reference - Mask group OR^3^ (95% CI): 0.59 (0.2-1.5) |

^1^Adjusted for age, sex, race/ethnicity, handwashing practices at baseline, sleep quality, stress, alcohol consumption, and flu vaccination
^2^Adjusted for gender, race, ethnicity, smoking status, physical activity, and having ever received a vaccination for influenza, intracluster correlation coefficient: -0.0005,
^3^Adjusted for age, sex, timely therapy of the index, vaccination of household contacts, time spent at home.
